# Effect of Interaction between Chromatin Loops on Cell-to-Cell Variability in Gene Expression

**DOI:** 10.1371/journal.pcbi.1004917

**Published:** 2016-05-06

**Authors:** Tuoqi Liu, Jiajun Zhang, Tianshou Zhou

**Affiliations:** 1 School of Mathematics and Computational Science, Sun Yat-Sen University, Guangzhou, People’s Republic of China; 2 Guangdong Province Key Laboratory of Computational Science, Sun Yat-Sen University, Guangzhou, People’s Republic of China; Rutgers University, UNITED STATES

## Abstract

According to recent experimental evidence, the interaction between chromatin loops, which can be characterized by three factors—connection pattern, distance between regulatory elements, and communication form, play an important role in determining the level of cell-to-cell variability in gene expression. These quantitative experiments call for a corresponding modeling effect that addresses the question of how changes in these factors affect variability at the expression level in a systematic rather than case-by-case fashion. Here we make such an effort, based on a mechanic model that maps three fundamental patterns for two interacting DNA loops into a 4–state model of stochastic transcription. We first show that in contrast to side-by-side loops, nested loops enhance mRNA expression and reduce expression noise whereas alternating loops have just opposite effects. Then, we compare effects of facilitated tracking and direct looping on gene expression. We find that the former performs better than the latter in controlling mean expression and in tuning expression noise, but this control or tuning is distance–dependent, remarkable for moderate loop lengths, and there is a limit loop length such that the difference in effect between two communication forms almost disappears. Our analysis and results justify the facilitated chromatin–looping hypothesis.

## Author Summary

The classic model of gene expression assumes direct spatial contact between a distal enhancer and a proximal promoter. Recent experimental evidence supported that enhancers and promoters are linked in a highly complex network of DNA–looping interactions, but it is unclear how these interactions including communication forms affect cell-to-cell variability in gene expression. Here we establish a quantitative model by mapping three possible patterns for two interacting DNA loops into a multistate model of gene expression. We first show that different connection patterns have different effects on gene expression, remarkable for short loop lengths. Then, we compare the results on the effects of two representative communication forms and find that the facilitated tracking performs better than the direct looping in controlling mean expression and in tuning expression noise, remarkable for moderate loop lengths. As such, we conclude that the former communication form is superior to the latter, rationalizing the facilitated DNA–looping hypothesis.

## Introduction

Regulatory elements and their interactions play a critical role in the spatial, temporal, and physiological control of gene expression [1,2]. These *cis*-acting elements can be divided into two distinct families: the one is composed of both the promoter and the regulatory elements with the distance from the transcription start site being less than 1kb [3,4], and the other contains the regulatory elements with the distance of much more than 1kb, which may be enhancers, silencers, insulators, or locus control regions [3]. Complexity of transcription activation is mainly in the ordered interactions among regulatory elements of the underlying gene, also including formation of DNA loops and interactions between DNA loops. These interactions are in essential biochemical, leading to stochastic gene transcription and further cell-to-cell variability in gene expression. Such stochasticity is essential for many cellular functions [5,6], and has been identified as a key factor underlying the observed phenotypic variability of genetically identical cells in homogeneous environments [7].

In general, transcription of genes is regulated by promoter–proximal DNA elements and distal DNA elements that together determine expression patterns of these genes. Remarkably in eukaryotic genomes, enhancers can be several hundreds of kilo–bases away from the promoter they regulate [8–10], and the intervening DNA can contain other promoters and other enhancers [11–14]. Enhancers activate promoters by directly contacting binding sites for transcription factors (TFs) and chromatin–modifying enzymes via DNA looping [15–19]. In theory, specific interactions between DNA elements can either assist enhancer–promoter looping by bringing the enhancer and the promoter closer together or are thought to interfere with enhancer–promoter looping by placing them in separate loop domains [20]. Moreover, these DNA elements may form chromatin loops in different manners, e.g., promoter–tethering elements in Drosophila that allow activation by specific enhancers over long distances are proposed to form DNA loops between sequences near the enhancer and the promoter [21,22]. Other examples include that in bacteriophage λ, the CI protein forms a 2.3-kb DNA loop that brings a distal stimulatory site close to RNA polymerase at the PRM promoter [23], and in the mouse β–globin locus, the Ldb1 protein appears to form a bridge necessary for efficient enhancer–promoter contact by binding to proteins at the locus control region and at the promoter [24]. To understand how a chromatin loop forms in a nucleus or how it is involved in gene regulation, Li, et al. [2] proposed that the major feature determining loop formation is the flexibility of chromatin, which may be modulated by histone acetylation and other modifications.

Single DNA looping and its functions have been intensively and extensively studied. For example, it has been shown that DNA looping can maintain a stable lysogenic state in the face of a range of challenges including noisy gene expression, nonspecific DNA binding, and operator site mutations [25], speed up the search process by bypassing proteins that block the sliding track close to the target [26], enhance lysogenic CI transcription in phage lambda [27], increase the range of bistability in a stochastic model of the lac genetic switch [28], and enhance or suppress transcriptional noise depending on conditions [29]. In addition, Boedicker, eta al. provided a quantitative characterization of the way that critical regulatory parameters modulate the output of transcriptional circuits involving DNA looping [30], Vilar, et al., showed that regulation based on DNA looping, in addition to increasing the repression level, can reduce the fluctuations of transcription and, at the same time, decrease the sensitivity to changes in the number of regulatory proteins [31], and Choudhary, et al., found that DNA loop formation is so fast that small bursts are averaged out, making it impossible to extract their size and frequency from the data [32]. In a word, previous studies that are restricted to analysis of single looping treated the looping mechanism as a local role, but according to the recent experimental evidence that reveals DNA–looping interactions, we currently recognize that the looping role is global (e.g., one DNA loop may interact with another DNA loop) [33].

The classic models of gene expression assume direct spatial contact between a distal enhancer and a proximal promoter [34–39]. However, recent chromatin capture technologies such as Chromosome Conformation Capture [40–41] have shown that enhancers and promoters are connected in a highly complex network of DNA–looping interactions [13,42,43]. For example, Wouter and his colleagues [44,45] showed that long–range (over tens or even hundreds of kilobases) gene regulation in eukaryotic cells involves spatial interactions between transcriptional elements, with intervening chromatin looping out. More interestingly, Priest, et al. [20], used two well–characterized DNA–looping proteins: Lac repressor and phage λ CI, to measure interactions between pairs of long DNA loops in *E*. *coli* cells in three possible topological arrangements of two pairs of interacting sites on DNA, namely, side-by-side loops, nested loops, and alternating loops. They found that the first loop pair does not affect each other; the second pair assists each other’s formation consistent with the distance–shortening effect; and the third pair, where one looping element is placed within the other DNA loop, inhibits each other’s formation, thus providing clear support for the loop domain model for insulation. They also argued that combining loop assistance and loop interference can provide strong specificity in long–range interactions. Another related yet important work is that Savitskaya, et al., experimentally observed [46] that when a pair of repressors and their binding sites are in between the enhancer and the promoter, the gene expression level is not lowered but is raised. For this counter–intuitive phenomenon, Mirny, et al., conjectured that this repressor pair shortens the distance between the enhancer and promoter pair, leading to the rise of the expression level [33].

**Fig 1:**
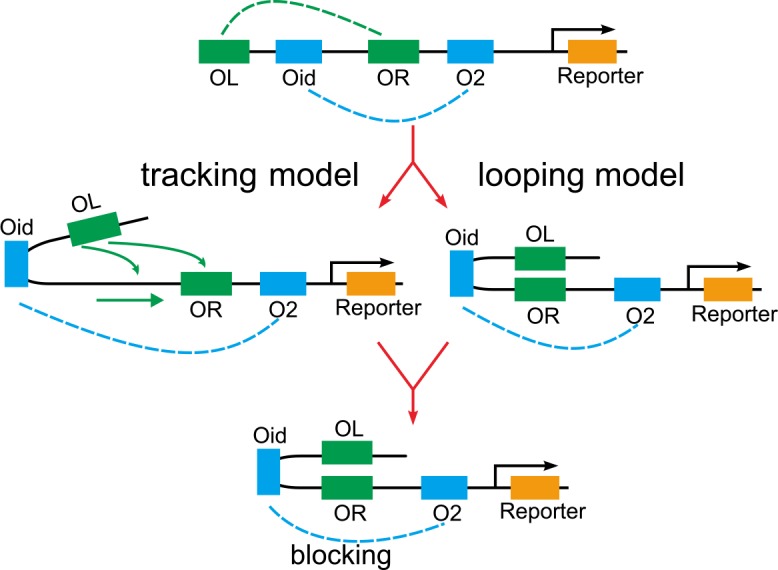
Schematic diagram for two representative forms that communication between enhancer and promoter takes: facilitated tracking and direct looping. It is unclear which form is more reasonable. This paper will justify the facilitated chromatin–looping hypothesis [2].

Another interesting conjecture is on communication between enhancers and promoters. The related questions are what form the enhancer–promoter communication takes (for example, what are the molecules that are transmitted between regulatory element and promoter), when this takes place, and whether this is the same for all classes of enhancers. This conjecture was put forward as early as in 1988 [47] and was later specified by Blackwood and Kadonaga [48] but has not been solved until now. Studies on specific loci, together with genome–wide approaches, suggest that there may be many common mechanisms involved in enhancer–promoter communication [1,49] (particularly see [49] wherein the author summarized 4 different kinds of communication models). In recent years, two views on communication between enhancer and promoter, namely the direct looping model and the facilitated–tracking model (referring to Fig **1**), have become the main stream or popular. The former model assumes a direct interaction between two chromosomal regions by looping out the intervening DNA sequence. For such communication mechanisms, various proteins have been proposed to bridge enhancers and promoters together as looped chromatin structures [50–52], and enhancer RNAs have also been proposed to be physically involved in establishing enhancer–promoter ‘looping’, and involving the integrator [53]. The latter model assumes that enhancer–bound proteins move progressively in a unidirectional manner towards the promoter, sometimes without leaving the enhancer sequence, thus resulting in the formation of a progressive loop that increases its size until it reaches the promoter to form a stable conformation. By modeling and analysis, we will justify the facilitated chromatin–looping hypothesis [1,2].

In a word, experiments have provided evidence for the influence of loop connection pattern, loop distance and communication form on gene expression, but how these factors impact cell-to-cell variability in gene expression remains not fully understood. We address this issue by developing on a stochastic model that maps connection patterns between interacting DNA loops into a multistate model of gene expression. In the case that communication form is not considered, we show that nested loops promote gene expression but reduce expression noise whereas alternating loops reduce the mean expression level but enlarge the noise, compared to side-by-side loops. In the case that communication form is considered, however, we find that the facilitated–tracking mechanism performs better than the direct looping mechanism in enhancing (or reducing) expression and reducing (or enlarging) noise, depending on connection patterns. Moreover, the effects of controlling expression and tuning noise are distance–dependent, remarkable for moderate loop lengths. In addition, we find that there is a limit loop length such that the difference in effect between two communication forms almost disappears. Our results imply that living organisms or cells would use the facilitated–tracking mechanism to deal with information about their environments.

## Methods

In order to reveal clearly the mechanism of how interacting DNA loops affects cell-to-cell variability in gene expression, here we consider only the case of two loops although multiple interacting loops may exist, remarkably in eukaryotic cells. We will establish the chemical master equation for the reaction network corresponding to each of three fundamental patterns, and use the binomial moment method that we previously developed [39] to solve this equation. We will focus on analyzing effects of connection pattern, loop length and communication form on mRNA levels.

### Hypotheses and settings based on experimental evidence

A pair of insulators Su and Hw found in the gypsy retrotransposon are the most potent enhancer blockers in the Drosophila melanogaster, but they do not prevent the enhancer–promoter communication, mainly because of their pairing interaction that results in mutual neutralization. Savitskaya, et al. [46], experimentally showed that long–distance functional interactions between Su and Hw can regulate communication between the enhancer and the promoter. Specifically, this insulator pair can interact with each other over considerable distances, across interposed enhancers or promoters and coding sequences, whereby enhancer blocking may be attenuated, cancelled, or restored. They also specified the role of the distance between these insulators in blocking enhancer–promoter communication.

Recall that Priest, et al. [20] measured interactions between pairs of long DNA loops in *E*. *coli* cells in three possible topological arrangements, using a pair of DNA–looping proteins (i.e., Lac repressor and phage *λ* CI). Thus, we can accordingly assume that the Su-Hw loop (called as the green loop in this paper) and the enhancer–promoter loop (the blue loop) interact with each other also in three connection patterns: alternating loops (called as the cross–type structure), nested loops (the inline–type structure), and side-by-side loops (the independence–type structure), although other connection patterns are possible in cases that space factors associated with DNA looping are considered. Furthermore, we assume that gene expression is enhanced if and only if the enhancer and the promoter form a loop, although it is possible that the enhancer–promoter loop represses gene expression. In addition, we assume that the fundamental production rate of mRNA is zero. In this paper, we do not consider the regulatory roles of TFs although they may influence chromatin looping and enhancer–promoter communication [1].

**Fig 2:**
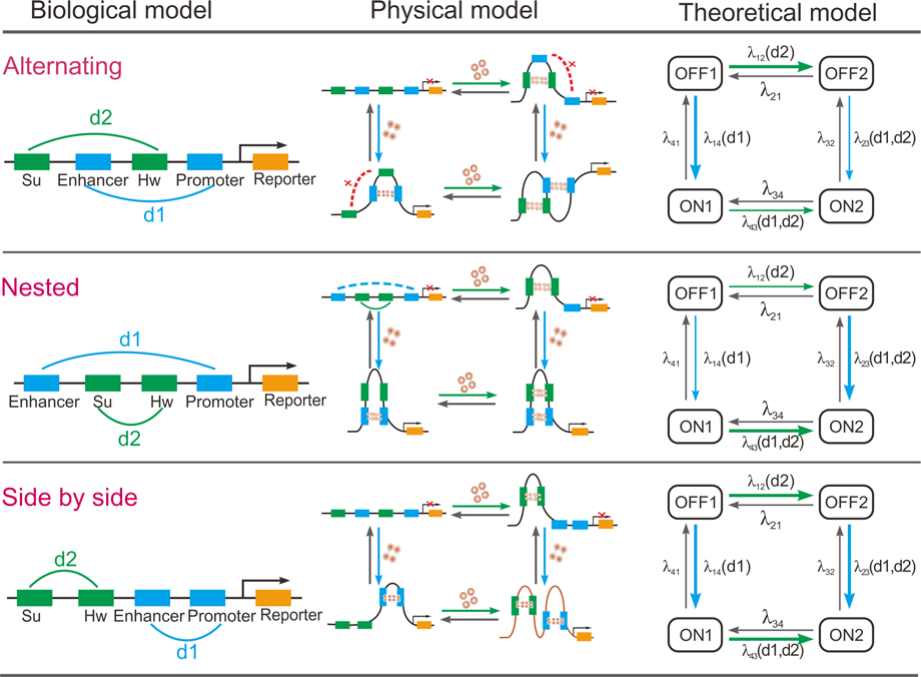
Modeling interactions between a pair of DNA loops: from physical models to biological models and to theoretical models. The blue and green loops influence gene expression in direct and indirect manners, respectively. The first column depicts fundamental biological structures for three kinds of interactions between two DNA loops, where the Su and Hw (green dock) may form a loop; the enhancer and promoter (blue dock) may form another loop. The second column depicts physical structures for respective DNA–looping interactions in the first column, which consider two different paths of looping (i.e., the Su and Hw pair or the enhancer and promoter pair is first looping). The third column represents respective theoretical models by mapping the physical models in the second column into a multistate model of gene expression, where transition rates between active and inactive states actually represent the looping rates, which depend on the loop lengths (along the DNA line), denoted by *d*_1_ for the blue loop but by *d*_2_ for the green loop.

Apart from connection patterns between interacting DNA loops, the rates of chromatin looping are also important factors affecting gene expression. In general, the looping rate for a pair of regulatory elements (e.g., Su and Hw) depends on not only the distance between this element pair but also the distances between other pairs of regulatory elements. In our case, related experimental results that support our model settings are stated as follows. In the alternating structure, the Su and Hw forms a blocking structure, which interferes with (referring to the red X in Fig **2**) the enhancer–promoter looping, thus reducing the looping rate and further decreasing gene expression. For example, if *λ* is the looping rate of enhancer and promoter, then this rate can be reduced to 0.3*λ* after its DNA loop is repressed by the other loop [20,46]. In the nested structure, the formation of the Su and Hw loop decreases the length of the enhancer–promoter loop, thus increasing the enhancer–promoter looping rate. For example, if *λ* is the looping rate of enhancer and promoter, then this rate can be increased to 8*λ* after its DNA loop is enhanced by the other loop [20,46]. In the side-by-side structure, the Su-Hw looping and the enhancer–promoter looping do not affect each other, so each has its looping rate. See Subsection 2.3 for more details.

Finally, although communication form between regulatory elements may be diverse, three representative mechanisms [1] have been proposed based on experimental evidence: the first mechanism is the linking model in which an activator protein first binds the promoter at a proximal sequence and facilitates the recruitment of a second TF at a site located just downstream of the former [54–56]. The second mechanism is the tracking model and/or a facilitated–tracking model in which histone acetylation and TF complexes are transiently detected in the intervening sequence and precedes transcription [52,57–59]. The third mechanism is the looping model that suggests a direct interaction between two chromosomal regions by looping out the intervening DNA sequence [53,60]. Currently, the latter two views have been extensively received, but it is unclear which communication mechanism is more possibly used by living organisms or cells. We will address this issue.

### Modeling interactions between two chromatin loops

First, the green loop formed by a pair of insulators (Su and Hw) and the blue loop formed by a pair of expression elements (enhancer and promoter) interact with each other in the one of three possible connection patterns: cross–type structure (due to alternating loops), inline–type structure (due to nested loops), and independence–type structure (due to side-by-side loops). Refer to the first column of schematic Fig **2**. For convenience, we denote by *d*_1_ the length of the blue loop along the DNA line, and by *d*_2_ the length of the green loop along the underlying DNA line. Experimental evidence supports that alternating loops give loop interference, nested loops give loop assistance, and side-by-side loops do not interact [20,46]. In spite of this, an unexplored question is how these interacting DNA loops affect gene expression (including mean outcome and expression noise).

In order to address this issue, we first introduce physical models for three fundamental patterns (alternating loops, nested loops and side-by-side loops), referring to the second column of Fig **2**. Note that in theory, the enhancer and promoter pair may form a loop but also may not form any loop, i.e., there are two possibilities. Similar cases are for the Su and Hw pair. Thus, there are in total four possibilities for each of three patterns. In order to help understanding, some details are listed below.

1. If both the blue loop and the green loop are formed, then the gene is expressed. However, the expression effect is different in the cases of cross–type and inline–type structures. Specifically, for the former, the formation of the green loop represses the effect that the blue loop enhances gene expression, whereas for the latter, the formation of the green loop has the just opposite effect.
2. If the blue loop is formed but the green loop is not formed, then the gene is also expressed. In this case, gene expression may be enhanced.
3. If neither the blue loop nor the green loop is formed, then the gene is not expressed.
4. If the blue loop is not formed but the green loop is formed, then the gene is not expressed either.

It is needed to point out that to derive the physical models shown in Fig **2**, we make simplifications, e.g., we do not consider binding of large protein complexes to promoters and enhancers, sequential recruitment of TFs to enhancers and promoters, and histone acetylation (and other modifications). All these factors or other complex processes would affect formation of DNA loops and interactions between them.

Then, we further map three physical models into a common multistate model of gene expression at the transcription level (the third column in Fig **2**). This mapping can bring us great conveniences for analysis and simulation. After mapping, the looping rates, which are functions of loop lengths, currently become transition rates between promoter activity states. Note that once two DNA loops are formed, any one of them can affect the length of the other, often in a nonlinear manner. This impact can lead to changes in transition rates and further in the gene outcome. Also note that the *ON*_1_ or *ON*_2_ state indicated in the theoretical model of Fig **2** means that the enhancer and the promoter form a loop (i.e., the blue loop) whereas the Su and the Hw may form a loop (i.e., the green loop) but also may not form any loop. In contrast, the *OFF*_1_ or *OFF*_2_ state means that the enhancer and the promoter do not form a loop whereas the Su and Hw pair may form a loop (i.e., the green loop) but also may not form any loop.

It is worth pointing out that the proposed–above mapping method is easily extended to cases of complex interactions among arbitrarily many pairs of chromatin loops.

### Dependence of looping rates on loop lengths

Note that looping rates are actually transition rates between on and off states shown in the third column of Fig **2** according to the above mapping relationships. In order to quantify how two interacting DNA loops affect the level of cell-to-cell variability in gene expression, it is needed to know the dependence of transition rates between active and inactive states in the mapped gene model on the lengths of these two loops (along the DNA) since different loop lengths would lead to different expression levels. For cases of single DNA loops, previous works studied the influence of the loop length on the looping rate, and even gave some experiential formulae between them [37,61]. In our case, these formulae read

$$\begin{array}{l} \lambda_{14}=k_{loop}^{\left(1\right)}=k_{R}^{on}\exp\left(-\frac{u}{d_{1}}-v\log\left(d_{1}\right)+wd_{1}+z\right)\\ \lambda_{12}=k_{loop}^{\left(2\right)}=k_{R}^{on}\exp\left(-\frac{u}{d_{2}}-v\log\left(d_{2}\right)+wd_{2}+z\right) \end{array}$$ (1)

where *k*_*loop*_ represents the rate of DNA looping, ad *d* is the loop length along the DNA line (i.e., the inter–operator distance). Some parameter values are set as $k_{R}^{on}=1$, *u* = 140.6, *v* = 2.52, *w* = 0.0014, and *z* = 19.9, which are obtained by fitting experimental data [37,61].

In general, for two interacting DNA loops, the rate of each DNA looping depends on not only its own loop length but also the length of the other loop, and is therefore a function of two variables. Note that parameter *λ*_23_ represents the looping rate of the blue loop after the green loop has been formed, implying that it is influenced by the former looping more than by the latter looping rate. Also note that the looping rate of the green loop relies on its own length *d*_2_. Therefore, *λ*_23_ is in principle a function of *d*_1_ and *d*_2_. Similarly, *λ*_43_ is also a function of *d*_1_ and *d*_2_. However, the existing experimental data only supported the quantitative relationship between the looping rate and the length of the blue loop [33]. Based on the above analysis and without loss of generality, we may set

$$\lambda_{23}=k_{1}\lambda_{14},\;\lambda_{43}=k_{2}\lambda_{12}$$ (2)

where the size of parameter *k* can determine the connection patterns of interacting DNA loops, according to Refs. [20,33]. In particular, according to experimental data [33] with small modifications, we may set

$$k=\{\begin{array}{cc} 4e^{-0.5d}+1>1 & \text{nested loops}\\ 1 & \text{side-by-side loops}\\ 0.5<1 & \text{alternating loops} \end{array}$$ (3)

where *d* represents the length of the underlying loop. First, this setting is reasonable since the formation of one loop in the nested, side-by-side, or alternating pattern enlarges, does not change, or reduces the length of the other loop, respectively. Second, the setting is based on observations from Fig 3A in Ref. [33] that for the nested pattern, k depends on the DNA loop distance in an exponential manner, whereas for the alternating pattern, the effect of reducing the loop length is remarkable when the blue loop distance is small but almost disappears when this distance is large.

Next, we consider the setting of *λ*_14_ and *λ*_12_ in the case that the so–called tracking (more precisely facilitated–tracking) mechanism between looping elements is considered. To help the reader understand this mechanism, we imagine a DNA loop as a string with some fixed length, two ends of which represent looping elements (e.g., Su and Hw). If one element slides along this string (here we only consider the sliding of one element in the green loop since we have assumed that the blue loop promotes gene expression), then this will affect the range that the enhancer and the promoter form the blue loop. Thus, the tracking mechanism leads to the increase in looping rates. Specifically, if we denote respectively by ${\tilde{\lambda }}_{14}$ and ${\tilde{\lambda }}_{12}$ the looping rates of the blue and green loops in the case that the facilitated–tracking mechanism is considered, and respectively by *λ*_14_ and *λ*_12_ the natural looping rates of these two loops, then we have

$${\tilde{\lambda }}_{14}=\lambda_{14}+\Delta_{1},\;{\tilde{\lambda }}_{12}=\lambda_{12}+\Delta_{2}$$ (4)

(correspondingly, *λ*_23_ and *λ*_34_ need to be modified). Note that for the facilitated–tracking mechanism, the longer the DNA loop length is, the more is the range that one regulatory element tracks another regulatory element, implying Δ∼*d*. Thus, it is reasonable to set Δ = *rd*, where *r* is a nonnegative parameter. Also note that no tracking or direct looping corresponds to *r* = 0 whereas tracking corresponds to *r* ≠ 0, so *r* characterizes the difference between the two mechanisms. Therefore, we call this parameter as the tracking ratio, which can be also understood as the probability that the enhancer and the promoter track to each other along the DNA line.

### Mathematical equations

In order to study qualitative and quantitative effects of the above three pairs of interacting DNA loops on gene expression (including the mean mRNA expression level and the mRNA noise intensity), we establish a mathematical model for the schematic diagram shown in the third column of Fig **2**. Assume that the gene has transcription only at *ON*_1_ to *ON*_2_ states with transcription rates denoted respectively by *μ*_1_ to *μ*_2_, and the mRNA degrades in a linear manner with the degradation rate denoted by *δ*. Let *λ*_14_ and *λ*_41_ stand respectively for transition rates from *OFF*_1_ to *ON*_1_ and vice versa, *λ*_12_ and *λ*_21_ respectively for transition rates from *OFF*_1_ to *OFF*_2_ and vice versa, *λ*_23_ and *λ*_32_ respectively for transition rates from *OFF*_2_ to *ON*_2_ and vice versa, and *λ*_34_ and *λ*_43_ respectively for transition rates from *ON*_1_ to *ON*_2_ and vice versa. Note that these transition rates are all functions of DNA loop lengths (along DNA lines). Denote by *P*_1_(*m*;*t*), *P*_2_(*m*;*t*), *P*_3_(*m*;*t*) and *P*_4_(*m*;*t*) the probabilities that the mRNA has *m* copies at *OFF*_1_, *OFF*_2_, *ON*_1_ and *ON*_2_ states respectively and at time *t*. Then, the chemical master equation for the full reaction system takes the following form

$$\begin{array}{l} \frac{\partial P_{1}\left(m;t\right)}{\partial t}=\lambda_{21}P_{2}\left(m;t\right)+\lambda_{41}P_{3}\left(m;t\right)-\left(\lambda_{14}+\lambda_{12}\right)P_{1}\left(m;t\right)+\delta \left(E-I\right)\left[mP_{1}\left(m;t\right)\right]\\ \frac{\partial P_{2}\left(m;t\right)}{\partial t}=\lambda_{12}P_{1}\left(m;t\right)+\lambda_{32}P_{4}\left(m;t\right)-\left(\lambda_{21}+\lambda_{23}\right)P_{2}\left(m;t\right)+\delta \left(E-I\right)\left[mP_{2}\left(m;t\right)\right]\\ \frac{\partial P_{3}\left(m;t\right)}{\partial t}=\lambda_{14}P_{1}\left(m;t\right)+\lambda_{34}P_{4}\left(m;t\right)-\left(\lambda_{41}+\lambda_{43}\right)P_{3}\left(m;t\right)+\mu_{1}\left(E^{-1}-I\right)\left[P_{3}\left(m;t\right)\right]+\delta \left(E-I\right)\left[mP_{3}\left(m;t\right)\right]\\ \frac{\partial P_{4}\left(m;t\right)}{\partial t}=\lambda_{23}P_{2}\left(m;t\right)+\lambda_{43}P_{3}\left(m;t\right)-\left(\lambda_{34}+\lambda_{32}\right)P_{4}\left(m;t\right)+\mu_{2}\left(E^{-1}-I\right)\left[P_{4}\left(m;t\right)\right]+\delta \left(E-I\right)\left[mP_{4}\left(m;t\right)\right] \end{array}$$ (5)

where *E* with the reverse *E*^−1^ is the step operator and *I* is the unit operator. In Eq (5), the quantitative dependences of all *λ* on loop lengths have been given in the previous subsection. It is worth pointing out that if more complex connection patterns for interactions between DNA loops are considered, then the corresponding chemical master equation can be generalized as [62]

$$\frac{dP\left(m;t\right)}{dt}=AP\left(m;t\right)+\Lambda \left(E^{-1}-I\right)\left[P\left(m;t\right)\right]+\delta \left(E-I\right)\left[mP\left(m;t\right)\right]$$ (6)

where **E** and **E**^−1^ are vectors of step operators and **I** is the vector of identity operators. In Eq (6), matrix **A** describes the interactions between chromatin loops, with the entries representing looping rates that are functions of loop lengths, diagonal matrix **Λ** describes the exits of transcription, with the diagonal elements representing transcription rates, and diagonal matrix **δ** depicts the degradation of mRNA, with the diagonal elements representing degradation rates.

### mRNA distribution and noise

It is in general difficult to solve a chemical master equation. However, the binomial moment approach that we developed previously [39] can well solve this question since it can conveniently give numerical solutions and even can give analytical solutions in some cases. In the following, we simply introduce this approach for the general gene model described by Eq (6). For this, we first introduce factorial binomial moments for factorial distributions *P*_*i*_(*m*;*t*), which are defined as

$$b_{k}^{\left(i\right)}=\sum_{m=k}^{\infty }\left(\begin{array}{c} m\\ k \end{array}\right)\;P_{i}\left(m;t\right),\;k=0,1,2,\cdots$$ (7)

Then, we can derive the following linear ordinary differential equations with constant coefficients from Eq (6)

$$\frac{d}{dt}b_{k}\left(t\right)=Ab_{k}\left(t\right)+\Lambda b_{k-1}\left(t\right)-\delta kb_{k}\left(t\right)$$ (8)

Our interest is in finding steady–state distribution. For convenience but without loss of generality, we assume that all the degradation rates are the same and the common degradation rate is 1. At steady state, factorial binomial moments satisfy the following algebraic equation

$$\left(-A+kI\right)b_{k}=\Lambda b_{k-1}$$ (9)

from which we obtain

$$b_{k}=\frac{1}{\prod_{i=1}^{k}\det\left(iI-A\right)}\prod_{i=k}^{1}\left[u_{N}{\left(iI-A\right)}^{*}\Lambda \right]\;b_{0}\begin{array}{cc} , & k=1,2,\cdots \end{array}$$ (10)

where $b_{k}=\sum_{i=1}^{N}b_{k}^{\left(i\right)}$ represents the total binomial moment with *k* called the order of this binomial moment, **u**_*N*_ = (1,1,⋯,1) is an *N*–dimensional row vector, and (*i***I** − **A**)* and det(*i***I** − **A**) are the adjacency matrix and the determinant of matrix (*i***I** − **A**), respectively. In Eq (10), the column vector ***b***_0_ is analytically given in S1 Text. Furthermore, we can use the following formula

$$P\left(m\right)=\sum_{k\geq m}{\left(-1\right)}^{m-k}\left(\begin{array}{c} k\\ m \end{array}\right)\;b_{k},\;m=0,1,2,\cdots$$ (11)

to reconstruct the steady–state mRNA distribution, where $P\left(m\right)=\sum_{i=1}^{N}P_{i}\left(m\right)$ represents the total distribution. In particular, we can conveniently use binomial moments to express the noise intensity defined as the ratio of variance over the square of mean. In fact, if this intensity is denoted by *η*_*m*_, then we have

$$\eta_{m}=\frac{2b_{2}+b_{1}-b_{1}^{2}}{b_{1}^{2}}$$ (12)

It is worth pointing out that formulae (11) and (12) are exact and can be directly used to numerical calculation and even derivation of analytical results. Note that the higher the orders of binomial moments are, the exacter are the resulting distributions calculated according to Eq (11) (S1 Text). In addition, the above formulae hold still in the dynamic case. In fact, we show numerical results on time evolutions and distributions of the mRNA number in Figs C–H in S1 Text, implying that our binomial moment approach can be also conveniently used to analysis of stochastic dynamics.

## Results

In this section, we will demonstrate qualitative and quantitative influences of three main factors underlying interactions between chromatin loops: connection pattern, loop length and communication form, on the gene outcome.

### Analytical distribution and noise

Here, we apply the above analysis to the model considered in this paper, i.e., to Eq (5). In our case, we have

$$b_{k}\left(t\right)=\left(\begin{array}{c} b_{k}^{\left(1\right)}\left(t\right)\\ b_{k}^{\left(2\right)}\left(t\right)\\ b_{k}^{\left(3\right)}\left(t\right)\\ b_{k}^{\left(4\right)}\left(t\right) \end{array}\right),\;A=\left(\begin{array}{cccc} -\left(\lambda_{14}+\lambda_{12}\right) & \lambda_{21} & \lambda_{41} & 0\\ \lambda_{12} & -\left(\lambda_{21}+\lambda_{23}\right) & 0 & \lambda_{32}\\ \lambda_{14} & 0 & -\left(\lambda_{41}+\lambda_{43}\right) & \lambda_{34}\\ 0 & \lambda_{23} & \lambda_{43} & -\left(\lambda_{32}+\lambda_{34}\right) \end{array}\right),\;\Lambda =\left(\begin{array}{cccc} 0 & 0 & 0 & 0\\ 0 & 0 & 0 & 0\\ 0 & 0 & \mu_{1} & 0\\ 0 & 0 & 0 & \mu_{2} \end{array}\right)$$

Assume that two transcription rates are the same, i.e., *μ*_1_ = *μ*_2_ = *μ*. Then, we can show from Eq (10) that the binomial moments have the following explicit expressions (S1 Text)

$$b_{k}={\tilde{b}}_{0}^{\left(3\right)}\frac{\mu^{k}}{k!}\prod_{i=1}^{k}\frac{\left(i+\gamma_{1}^{\left(1\right)}\right)\left(i+\gamma_{2}^{\left(1\right)}\right)\left(i+\gamma_{3}^{\left(1\right)}\right)}{\left(i+\alpha_{1}\right)\left(i+\alpha_{2}\right)\left(i+\alpha_{3}\right)}+{\tilde{b}}_{0}^{\left(4\right)}\frac{\mu^{k}}{k!}\prod_{i=1}^{k}\frac{\left(i+\gamma_{1}^{\left(2\right)}\right)\left(i+\gamma_{2}^{\left(2\right)}\right)\left(i+\gamma_{3}^{\left(2\right)}\right)}{\left(i+\alpha_{1}\right)\left(i+\alpha_{2}\right)\left(i+\alpha_{3}\right)}$$ (13)

where all *γ* are constants depending on system parameters (we omit their concrete expressions due to complexity), ${\tilde{b}}_{0}^{\left(i\right)}=b_{0}^{\left(i\right)}/\left(b_{0}^{\left(3\right)}+b_{0}^{\left(4\right)}\right),i=3,4,-\alpha_{1},-\alpha_{2},-\alpha_{3}$ are nonzero characteristic values of M–matrix **A**, and $b_{0}^{\left(k\right)}=\prod_{i=1}^{3}\left(\beta_{i}^{\left(k\right)}/\alpha_{i}\right),\;1\leq k\leq 4$with $-\beta_{1}^{\left(k\right)},-\beta_{2}^{\left(k\right)},\cdots ,-\beta_{N-1}^{\left(k\right)}$ being nonzero characteristic values of matrix **M**_*k*_ (the minor of the element *a*_*kk*_ of matrix **A**). To that end, using Eq (11), we can further arrive at the following analytical expression of mRNA distribution

$$P\left(m\right)={\tilde{b}}_{0}^{\left(3\right)}P_{1}\left(m\right)+{\tilde{b}}_{0}^{\left(4\right)}P_{2}\left(m\right)$$ (14)

where

Pi(m)=μmm!(γ1(i))m(γ2(i))m(γ3(i))m(α1)m(α2)m(α3)mF33(m+γ1(i)m+γ2(i)m+γ3(i)m+α1m+α2m+α3|;−μ),i=1,2 (15)

in which Fnn(a1⋯anb1⋯bn|;z) is a confluent hypergeometric function [62,63], and (*c*)_*n*_ is the Pochhammer symbol and defined as (*c*)_*n*_ = Γ(*c*+*n*)/Γ(*c*) with Γ(*c*) being the common Gamma function. Eq (14) indicates that the mRNA distribution is a linear combination of two distributions with each similar to that in the common two–state gene model at the transcription level [62]. Correspondingly, the mRNA noise intensity is given by Eq (12) with

$$b_{1}=\frac{\mu \left[C\left(1\right)b_{0}^{\left(3\right)}+D\left(1\right)b_{0}^{\left(4\right)}\right]}{\left(1+\alpha_{1}\right)\left(1+\alpha_{2}\right)\left(1+\alpha_{3}\right)},\;b_{2}=\frac{\mu^{2}}{2}\frac{\left[C\left(2\right)W_{33}^{\left(1\right)}+D\left(2\right)W_{34}^{\left(1\right)}\right]b_{0}^{\left(3\right)}+\left[C\left(2\right)W_{43}^{\left(1\right)}+D\left(2\right)W_{44}^{\left(1\right)}\right]b_{0}^{\left(4\right)}}{\left(1+\alpha_{1}\right)\left(i+\alpha_{2}\right)\left(1+\alpha_{3}\right)\left(2+\alpha_{1}\right)\left(2+\alpha_{2}\right)\left(2+\alpha_{3}\right)}$$ (16)

where *C*(*i*), *D*(*i*) and all *W* are given in S1 Text. Note that Eqs (14)–(16) are exact for any values of the system parameters, and hence can be directly used to numerical calculations.

The above general yet exact results can be simplified in some cases. For example, according to experimental evidence [20,33], we can set *λ*_14_ = *λ*_23_, *λ*_12_ = *λ*_34_, *λ*_41_ = *λ*_32_, and *λ*_21_ = *λ*_43_ for the side-by-side pattern of interacting DNA loops; 0.5*λ*_14_ = *λ*_23_, 0.5*λ*_12_ = *λ*_43_, *λ*_41_ = *λ*_32_, and *λ*_21_ = *λ*_34_ for the alternating pattern; and (4*e*^−0.5*d*^ + 1)*λ*_14_ = *λ*_23_, (4*e*^−0.5*d*^ + 1)*λ*_12_ = *λ*_43_, *λ*_41_ = *λ*_32_, and *λ*_21_ = *λ*_34_ for the nested pattern. In the following, we consider two particular cases. First, if *λ*_23_ = *λ*_43_ = *kλ* (*k* is a positive constant but possibly depends on DNA loop lengths along the DNA lines) and *λ*_12_ = *λ*_14_ = *λ*_21_ = *λ*_34_ = *λ*_32_ = *λ*_41_ = *λ*, then it is interesting that we find that for the side-by-side pattern, the steady–state mRNA number follows a distribution of the form (S1 Text)

P(m)=μ˜mm!(a1)m(a2)m(b1)m(b2)mF22(m+a1m+a2m+b1m+b2|;−μ˜) (17)

where $\tilde{\mu }$, *a*_*i*_ and *b*_*i*_ are constants depending on system parameters including parameter *k*, and in particular, they are functions of two transition rates *λ*_12_ and *λ*_14_, each that is further functions of DNA loop lengths given by Eq (1) but may take an arbitrarily positive value. This distribution is in accord with our intuition since two DNA loops do not interact to each other, which leads to the bursting expression of the gene in a manner similar to that in the common two–state gene model at the transcription level. The corresponding noise intensity is given by

$$\eta_{m}=\left[\frac{\left(1+a_{1}\right)\left(1+a_{2}\right)}{\left(1+b_{1}\right)\left(1+b_{2}\right)}+\frac{1}{\tilde{\mu }}\right]\frac{b_{1}b_{2}}{\tilde{\mu }a_{1}a_{2}}-1$$ (18)

Next, we consider an extreme case, i.e., all the transition rates are the same (this is possible only for the side-by-side pattern). In this case, we find that the mRNA number follows a simpler distribution of the form (also S1 Text)

P(m)=μmm!(λ)m(2λ)mF11(m+λ,m+2λ;−μ) (19)

The corresponding noise intensity is reduced to

$$\eta_{m}=\frac{1}{4\left(1+2\lambda \right)}+\frac{2}{\mu }$$ (20)

### Effects of connection pattern and loop length on gene expression

The above analytical results in principle show how three connection patterns and two loop lengths as well as tracking probability or tracking ratio (characterized by parameter *r*) impact gene expression (including mRNA distribution, mean expression and noise intensity), but the results are implicit and not intuitive. Here, we perform numerical calculation to give intuitive results for this impact. Since the output level is directly related to the length of the blue loop (along the DNA line), we first let this length change but keep the green loop length fixed. Also since our focus is on effects of connection pattern and loop length on gene expression, we do not consider the impact of the communication mechanism (i.e., we do not consider tracking between loop elements) in this subsection, and will leave it to the next subsection for investigation.

**Fig 3:**
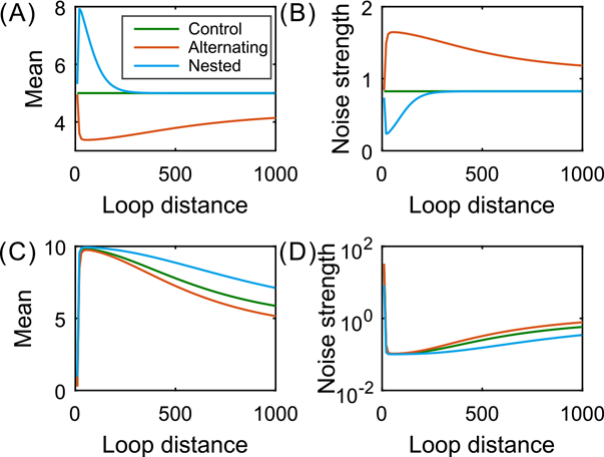
**Shown is the dependence of mean expression (A, B) and expression noise (C, D) on the blue loop length (along the DNA line) for three fundamental patterns: alternating loops, nested loops and side-by-side loops.** Here the side-by-side structure is taken as a control or reference since the blue loop length does not affect the expression level nor the noise intensity (see green lines). In all cases, parameter values are set as *μ* = 10, *δ* = 1, *r* = 0, *d*_2_ = 1500, *λ*_21_ = *λ*_32_ = *λ*_34_ = *λ*_41_ = 0.3, *k*_2_ = 0.5, and $k_{1}=4e^{-0.5d_{1}}+1$ with *d*_1_ ∈ (0,1000).

Figs **3C** and **2D** show how the mean expression level and the mRNA noise intensity depend on the blue loop length for three connection patterns: alternating loops, nested loops and side-by-side loops, where the green loop length is fixed at 1500 whereas the blue loop length changes in the interval (0,1000). We observe that the mean level in each of the three structures is fundamentally a monotonically decreasing function of the blue loop length whereas the noise intensity is fundamentally a monotonically increasing function of this length, except for a small range of the loop length where both have local extreme values (more specifically, the mean expression level has a local maximum at a certain value of the DNA loop length whereas the noise intensity has a local minimum at another value of the length). Moreover, the mean level is generally lower in the case of alternating loops or higher in the case of nested loops than in the case of side-by-side loops, implying that the cross–type structure reduces the mean expression whereas the inline–type structure enlarges the mean level. On contrary, the noise intensity is generally higher in the case of alternating loops or lower in the case of nested loops than in the case of side-by-side loops, implying that the cross–type structure enlarges the expression noise whereas the inline–type structure reduces the expression noise. From these results, however, one cannot clearly see qualitative differences in the effect of the blue loop length on gene expression between alternating and the nested structures.

Since two loops in the side-by-side structure are independent of each other or since the green loop does not influence the blue loop length that is directly related to the expression efficiency, we then take the side-by-side structure as a reference or control to show the effects of alternating loops and nested loops on gene expression, respectively. Fig **3A** and **3B** show how the mean expression level and the noise depend on the blue loop length. From these two diagrams, we observe that the nested structure increases the mean expression level but reduces the expression noise. In contrast, the alternating structure decreases the mean expression level but enlarges the expression noise. We also observe that the effects are most remarkable in the cases of small blue loop lengths. All these observations are in good accord with experimental observations [20,46] and also with our intuition. This is because in the alternating structure, the looping of Su and Hw intervenes the formation of the enhancer–promoter loop, thus reducing the latter looping rate and further weakening gene expression. In the nested structure, the Su and the Hw form a loop, which decreases the length of the enhancer–promoter loop but increases the looping rate of enhancer and promoter.

In addition, we demonstrate numerical results on time evolutions and distributions of the mRNA number in S1 Text, referring to Figs C–F in S1 Text wherein more numerical results are shown. This demonstration has two aims: the one is to show that the results predicted by theory are in accord with those obtained by the Gillespie stochastic simulation algorithm; the other is to show that our binomial moment approach can be easily used to analysis of transient dynamics.

### Influence of communication form on gene expression

After having understood the fundamental functions of alternating and nested structures, we now turn to considering the effects of the forms that the enhancer–promoter communication takes on gene expression. By comparing differences in effects induced by direct looping and facilitated tracking between these two connection patterns, we try to answer the question of which of these two representative mechanisms is more reasonable or more possibly used by living organisms.

In the previous subsection, we considered the case that the blue loop length changes but the green loop length is fixed. In this subsection, we will consider the case that the green loop length changes but the blue loop length is fixed. This consideration is mainly for comparing qualitatively different effects of direct looping (i.e., no tracking, characterized by *r* = 0) and facilitated tracking (characterized by 0 < *r* ≤ 1) mechanisms on gene expression.

First, we consider the case of the alternating structure. Note that in this structure, the Su and Hw pair indirectly represses gene expression by negatively influencing the looping of enhancer and promoter. Since parameter *r* can represent the probability that one DNA element tracks the other DNA element, we call it as the tracking ratio. Also note that for this cross–type structure, the larger the size of *r* is, the more difficult is the enhancer–promoter looping, implying that gene expression is weakened. In addition, note that for the alternating loop pattern, the tracking of looping elements is limited, and this limitation implies that the role that the Su-Hw looping weakens indirectly gene expression is also limited.

**Fig 4:**
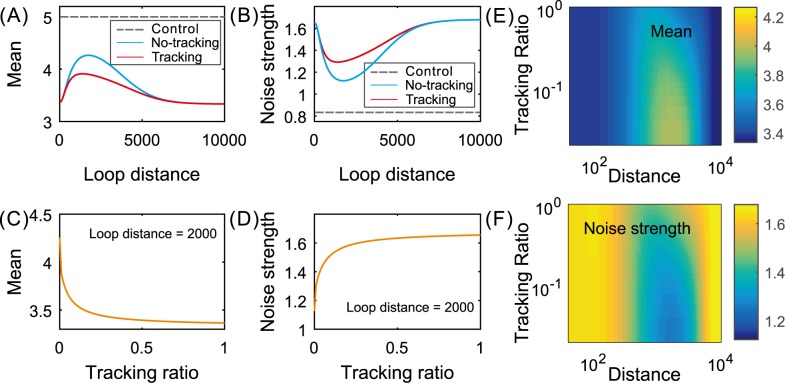
Comparison of effects between the cases of no tracking (*r* = 0) and tracking (positive *r*): cross–type structure. (A,B) dependence of mean expression and noise intensity on the green loop length for a fixed tracking ratio (*r* = 0.1), where the dashed lines correspond to the side-by-side loops; (C,D) dependence of mean expression and noise intensity on tracking ratio for a fixed green loop length; (E,F) three–dimensional pseudo diagrams for dependence of the mean expression level/the noise intensity on both the loop length and the tracking ratio, where the color change in the bar represents the change in the mean level or in the noise. Parameter values are set as *d*_1_ = 1500, *μ* = 10, *δ* = 1, *r* = 0.1, *λ*_21_ = *λ*_32_ = *λ*_34_ = *λ*_41_ = 0.3, *k*_1_ = 0.5, and $k_{2}=4e^{-0.5d_{2}}+1$ with *d*_2_ ∈ (0,10000) in (A) and (B) but *k*_2_ = 0.5 in (C) and (D).

In order to show the effect of the communication mechanism on gene expression in the case of the cross–type structure, we first compare the difference in effect between the cases: tracking exits (*r* = 0.1) and no tracking exits (*r* = 0), referring to Fig **4A** and **4B**. We observe that the mean level in the case of tracking is in general lower than that in the case of no tracking but the noise intensity is in general higher in the former case than in the latter case. Then, we show the dependences of the mean level and the noise intensity on the tracking ratio for a fixed green loop length, referring to Fig **4C** and **4D**. From these two diagrams, we observe that the mean level is a monotonically decreasing function whereas the noise intensity is a monotonically increasing function. The monotonicity becomes more apparent for small tracking ratios but disappears when the tracking ratio is beyond a threshold. Fig **4E** or **4F** shows a panoramic view for how the mean expression level or the noise intensity depends both on the green loop distance and on the tracking ratio. We observe that (1) only for moderate loop distances, does the mean expression level or the noise intensity have apparent differences between tracking and no–tracking cases; (2) the dependence of the mean level on both the loop length and the tracking ratio is fundamentally opposite to that of the noise intensity on both the loop length and the tracking ratio. These results are in accord with those shown in Fig **4A**–**4D**.

Then, we consider the case of the inline–type structure. Note that in this structure, the Su and Hw pair indirectly represses gene expression by positively influencing the looping of enhancer and promoter. Also note that for this structure, the larger the size of *r* is, the enhancer and the promoter form a loop more easily (just opposite to the case of the cross–type structure), implying that the gene expression is promoted since we have assumed that the enhancer–promoter looping promotes gene expression. In addition, note that for the nested loop pattern, the tracking between looping elements has no limitation, implying that the role of what the Su-Hw looping enhances directly gene expression is further promoted with increasing the size of *r*.

**Fig 5:**
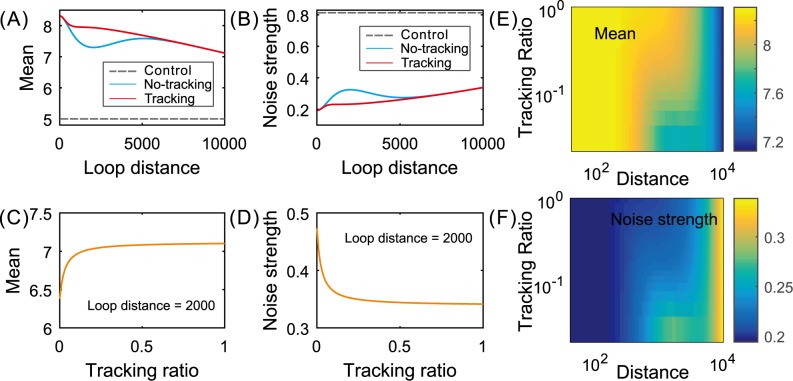
Comparison of effects between the cases of tracking (*r* > 0) and non–tracking (*r* = 0): inline–type structure. (A,B) dependence of mean expression and noise intensity on the green loop length for a fixed tracking ratio (*r* = 0.1), where the dashed lines correspond to the side-by-side loops; (C,D) dependence of mean expression and noise intensity on tracking ratio for a fixed green loop length; (E,F) three–dimensional pseudo diagrams for dependence of the mean expression level/the noise intensity on both the loop length and the tracking ratio, where the color change in the bar represents the change in the mean level or in the noise. Parameter values are set as *d*_1_ = 1500, *λ*_21_ = *λ*_32_ = *λ*_34_ = *λ*_41_ = 0.3, *μ* = 10, *δ* = 1, *r* = 0.1, $k_{1}=k_{2}=4e^{-0.5d_{2}}+1$ with *d*_2_ ∈ (0,10000) in (A) and (B) but $k_{1}=k_{2}=4e^{-0.5d_{1}}+1$ with *d*_1_ ∈ (0,10000) in (C) and (D).

In addition, we use Fig **5E** or **5F** to show a panoramic view for how the mean expression level or the noise intensity depends on both the green loop distance and the tracking ratio. Similar to the case of the cross–type structure, we still observe the following two points: (1) only for moderate loop distances, does the mean expression level or the noise intensity have apparent differences between tracking and no–tracking cases; (2) the dependence of the mean level on both the loop length and the tracking ratio is fundamentally opposite to that of the noise intensity on both the loop length and the tracking ratio. These results are in accord with those shown in Fig **5A**–**5D**.

In order to show the effect of communication mechanisms on gene expression in the case of the inline–type structure, we first compare the difference in effect between the cases that tracking exits (*r* = 0.1) and no tracking exits (*r* = 0), referring to Fig **5A** and **5B**. We observe that the mean level in the case of tracking is lower than that in the case of no tracking but the noise intensity is higher in the former case than in the latter case. Then, we show the dependences of the mean level and the noise intensity on the tracking ratio for a fixed green loop length, referring to Fig **5C** and **5D**. From these two diagrams, we observe that the mean level is a monotonically increasing function whereas the noise intensity is a monotonically decreasing function. This monotonicity becomes more apparent for small tracking ratios, but finally disappears when the tracking ratio is beyond a threshold.

**Fig 6:**
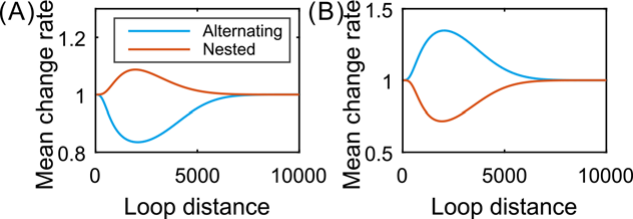
**Dependence of relative change ratios on the green loop length: (A) mean expression and (B) noise intensity.** Here, parameter values are set as *d*_1_ = 1500, *λ*_21_ = *λ*_32_ = *λ*_34_ = *λ*_41_ = 0.3, *μ* = 10, *δ* = 1, *r* = 0.15, and *k*_1_ = *k*_2_ = 0.5 for alternating loops but $k_{1}=k_{2}=4e^{-0.5d_{2}}+1$ with *d*_2_ ∈ (40,10000) for nested loops.

In addition, we use Fig **6** to show the quantitative dependence of the relative change ratio that is defined as the ratio of the difference between the mean expression level (or the noise intensity) in the tracking case and that in the non–tracking case over the mean expression level (or the noise intensity) in the non–tracking case, on the green loop length. We observe that for the inline–type structure, the relative change ratio of the mean expression is more than zero whereas that of the noise intensity is less than zero, remarkably for moderate lengths of the Su-Hw loop. For the cross–type structure, however, this ratio of the mean expression is less than or equal to zero whereas that of the noise intensity is equal to or greater than zero, remarkably for moderate lengths of the green loop. In particular, the largest relative change ratio for the mean expression is 2% lower in the non–tracking case than in the tracking case for the alternating structure, whereas it is 4% higher in the former case than in the latter case for the nested structure. In a word, the results shown in Fig **6** indicate that nested loops play a role of enhancing gene expression and reducing noise while alternating loops play a role of repressing gene expression and enlarging expression noise. Moreover, there is a limit length of the green loop such that the effect of this control almost disappears.

The combination of Figs **4**–**6** implies that from the viewpoint of controlling gene expression, the facilitated–tracking communication (i.e., *r* ≠ 0) is advantageous over the direct–looping communication (i.e., *r* = 0), independent of connection patterns between interacting DNA loops. In addition, there is an optimal loop length such that the mean expression level or the expression noise intensity is maximal or minimal. This is an interesting phenomenon. From the viewpoint of mathematics, the occurrence of this phenomenon is generated because the looping rates are nonlinear functions of loop lengths along DNA lines. From the viewpoint of biology, however, this implies that if two loop elements are very close or very far away, then the expression level is low, and that there is a suitable location of these two loop elements such that the expression level reaches the highest value.

In order to show effects of the number of interacting DNA loops on the obtained-above qualitative results, we also investigate three interacting chromatin loops in Fig M in S1 Text, and find that the qualitative results are invariant.

## Discussion

We have studied the influence of interactions between DNA loops on gene expression, using a quantitative model established by mapping fundamental structures of DNA–looping interactions into a multistate model of stochastic gene expression. In contrast to previous gene models that assume direct spatial contact between a distal enhancer and a proximal promoter [34–38], our model has the advantage in many aspects, e.g., three main factors (connection pattern, loop length and communication form) characterizing DNA–looping interactions are easily incorporated in the model (even including those in more complex cases). Also for example, it can be analytically solved using the binomial moment method that we previously developed [39], with results that can well show how key yet experimentally testable parameters associated with the interactions between DNA loops such as looping rates, loop lengths and the tracking ratio affect gene expression levels. Thus, our model provides a possible platform for experimentally testing effects of specific connection patterns, loop lengths and communication forms on gene expression. In fact, we have found good agreement between results obtained by our model and experimental measurements [20,46].

We have analyzed effects of three fundamental structures of DNA–looping interactions (alternating loops, nested loops, and side-by-side loops) on gene expression including the mean level and the output noise, and shown that different structures have different effects. However, as pointed out in the introduction, enhancers and promoters may be connected in a highly complex network of DNA–looping interactions [13,42,43], remarkably in eukaryotic cells. One main question that needs to be addressed is at which step during gene activation, various nucleoprotein complexes assemble at distant enhancers, and how these complexes then contribute to promoter accessibility, the preinitiation complex recruitment and/or assembly, and transcription initiation and elongation. Enhancers have been shown to have a role in the preinitiation complex recruitment at target promoters [64–68], the removal of proteasome complexes at promoters [69], the generation of intra-chromosomal loops between regulatory regions [70], and the regulation of elongation [71–75]. Enhancers are also involved in the removal of repressive histone modifications [76–79], suggesting that they also contribute to the delivery of enzymes that regulate histone modifications [80]. In a word, enhancers in eukaryotic genomes can be many hundreds of kilobases away from the promoter they regulate [8–10], and the intervening DNA can contain other promoters and other enhancers [11–14]. For these complex cases, how the regulatory influence of distal elements is exerted efficiently and specifically at the correct promoters is worth further investigation.

In this paper, we have also shown the influences of two representative mechanisms of loop element tracking (namely direct looping and facilitated tracking) on gene expression. By comparing their respective performance in controlling mean expression and in tuning expression noise, we conclude that from the perspective of controlling gene expression and tuning expression noise, the facilitated tracking is superior to the direct looping, remarkably for moderate loop lengths. In spite of this, a question is whether increasing the enhancer–promoter distance in higher organisms favors particular mechanisms of interactions between regulatory elements, for example, looping rather than tracking or linking. In fact, given a larger distance (>10 kb and up to 100s of kb) separating many enhancers from their target promoter, it is difficult to envisage a mechanism in which the intervening chromatin is directly involved in a mechanism of enhancer–promoter communication. Therefore, tracking mechanisms are likely limited to enhancers that are close (1–10 kb) to their target promoters. In addition, the stiffness of the chromatin fibre might restrict short–range enhancer–promoter interactions, with a minimal estimated length of 10 kb for uninterrupted 30-nm chromatin fibres and 0.5 kb for naked DNA [71,81,82]. In a more general sense, genes transcribed by RNA polymerase have two distinct families of cis-acting elements: the promoter and more remote cis-regulatory elements. The former, the length of which is generally less than 1kb, is composed of a core promoter [83,84], whereas the latter, the length of which is in general more than 1kb, can be enhancers, silencers, insulators, or locus control regions [3]. The exact composition of core promoter elements may be a key determinant of enhancer–promoter specificity [85,86]. These regulatory elements may communicate to each another in different manners [1]. When various possible regulatory elements and the distances among them are simultaneously considered, how the corresponding communication mechanisms affect gene expression still remains poorly understood and further studies are required.

Finally, although numerous studies in several multi–gene clusters have shown that gene activation by a remote enhancer is associated with chromatin loop formation [87,88], the nature of its formation and involvement in gene regulation is not fully understood. Li, el al. [2], proposed that the major feature that determines loop formation is the chromatin flexibility, which is modulated by histone acetylation (and other modifications). Based on this, they also concluded that histone modifications lead to the determination of the probability of interaction between a remote enhancer and its cognate genes, and these features constitute the main elements of the facilitated chromatin–looping hypothesis. Our model analysis has partially verified reasonability of this hypothesis, but relevant experimental tests are required.

## Supporting Information

S1 Text It consists of two parts: Derivation of analytical results and Supplementary numerical results. The former gives details for derivation of mathematical formulae used in the main text. The latter contains the following contents: (1) Three–dimensional effects for dependence of the mRNA mean/the mRNA noise on loop distance and tracking ratio; (2) Time evolution and distribution of the mRNA number; (3) Skewness and kurtosis of the mRNA distribution; (4) Effect of different transcript rates on gene expression; Effect of three interacting DNA loops on gene expression.
(DOCX)

## References

[1] Vernimmen D, Bickmore WA (2015). The hierarchy of transcriptional activation: From enhancer to promoter. Trends in Genet 31(12): 696–708. 26599498 10.1016/j.tig.2015.10.004

[2] Li QL, Barkess G, Qian H (2006). Chromatin looping and the probability of transcription. Trends in Genet 22 (4): 197–202. 16494964 10.1016/j.tig.2006.02.004

[3] Ong CT, Corces VG (2011) Enhancer function: new insights into the regulation of tissue-specific gene expression. Nat Rev Genet 12: 283–293. 10.1038/nrg2957 21358745 PMC3175006

[4] Lenhard B, Sandelin A, Piero Carninci P (2012). Metazoan promoters: emerging characteristics and insights into transcriptional regulation. Nat Rev Genet 13: 233–245. 10.1038/nrg3163 22392219

[5] Boettiger AN, and Levine M (2009). Synchronous and stochastic patterns of gene activation in the early Drosophila embryo. Science 325: 471–473. 10.1126/science.1173976 19628867 PMC4280267

[6] Raj A, Rifkin SA, Andersen E, van Oudenaarden A (2010). Variability in gene expression underlies incomplete penetrance. Nature 463, 913–918. 10.1038/nature08781 20164922 PMC2836165

[7] Eldar A, and Elowitz MB (2010). Functional roles for noise in genetic circuits. Nature 467: 167–173. 10.1038/nature09326 20829787 PMC4100692

[8] Letticea LA, Horikoshi T, Heaneya SJH, van Baren MJ, van der Linde HC, et al (2002). Disruption of a long-range cis-acting regulator for Shh causes preaxial polydactyly. Proc Natl Acad Sci USA 99(11):7548–7553. 12032320 10.1073/pnas.112212199 PMC124279

[9] Nobrega MA, Ovcharenko I, Afzal V, Rubin EM (2003). Scanning human gene deserts for long-range enhancers. Science 302(5644):413 14563999 10.1126/science.1088328

[10] Jin F, Li Y, Dixon JR, Selvaraj S, Ye Z, et al (2013). A high-resolution map of the three-dimensional chromatin interactome in human cells. Nature 503(7475):290–294. 10.1038/nature12644 24141950 PMC3838900

[11] Maeda RK, Karch F (2003) Ensuring enhancer fidelity. Nat Genet 34(4):360–361. 12923541 10.1038/ng0803-360

[12] Li G, Ruan XA, Raymond K, Auerbach RK, Sandhu KS, et al (2012). Extensive promoter-centered chromatin interactions provide a topological basis for transcription regulation. Cell 148(1–2):84–98. 10.1016/j.cell.2011.12.014 22265404 PMC3339270

[13] Kieffer-Kwon K-R, Tang ZH, Mathe E, Qian J, Sung MH, et al (2013) Int.eractome maps of mouse gene regulatory domains reveal basic principles of transcriptional regulation. Cell 155(7):1507–1520. 10.1016/j.cell.2013.11.039 24360274 PMC3905448

[14] Marinic M, Aktas T, Ruf S, Spitz F (2013). An integrated holo-enhancer unit defines tissue and gene specificity of the Fgf8 regulatory landscape. Dev Cell 24(5): 530–542. 10.1016/j.devcel.2013.01.025 23453598

[15] Ptashne M (1986). Gene regulation by proteins acting nearby and at a distance. Nature 322(6081):697–701. 3018583 10.1038/322697a0

[16] Tolhuis B, Palstra RJ, Splinter E, Grosveld F, de Laat W (2002). Looping and interaction between hypersensitive sites in the active beta-globin locus. Mol Cell 10(6):1453–1465. 12504019 10.1016/s1097-2765(02)00781-5

[17] Carter D, Chakalova L, Osborne CS, Dai YF, Fraser P (2002) Long-range chromatin regulatory interactions in vivo. Nat Genet 32(4):623–626. 12426570 10.1038/ng1051

[18] Bulger M, Groudine M (2011) Functional and mechanistic diversity of distal transcription enhancers. Cell 144(3):327–339. 10.1016/j.cell.2011.01.024 21295696 PMC3742076

[19] Krivega I, Dean A (2012) Enhancer and promoter interactions-long distance calls. Curr Opin Genet Dev 22(2):79–85. 10.1016/j.gde.2011.11.001 22169023 PMC3342482

[20] Priesta DG, Kumar S, Yan Y, Dunlap DD, Dodda IB, Shearwin KE (2014). Quantitation of interactions between two DNA loops demonstrates loop domain insulation in E. coli cells. Proc Natl Acad Sci USA 111(42): E4449–E4457. 10.1073/pnas.1410764111 25288735 PMC4210295

[21] Kwon D, Mucci D, Langlais KK, Americo JL, DeVido SK, et al (2009) Enhancer–promoter communication at the Drosophila engrailed locus. Development 136(18):3067–3075. 10.1242/dev.036426 19675130 PMC2730364

[22] Calhoun VC, Stathopoulos A, Levine M (2002) Promoter-proximal tethering elements regulate enhancer–promoter specificity in the Drosophila Antennapedia complex. Proc Natl Acad Sci USA 99(14):9243–9247. 12093913 10.1073/pnas.142291299 PMC123125

[23] Cui L, Murchland I, Shearwin KE, Dodd IB (2013) Enhancer-like long-range transcriptional activation by λ CI-mediated DNA looping. Proc Natl Acad Sci USA 110(8):2922–2927. 10.1073/pnas.1221322110 23382214 PMC3581938

[24] Deng W, Lee J, Wang HX, Miller J, Reik A, et al (2012) Controlling long-range genomic interactions at a native locus by targeted tethering of a looping factor. Cell 149(6):1233–1244. 10.1016/j.cell.2012.03.051 22682246 PMC3372860

[25] Morelli MJ, ten Wolde PR, Allen RJ (2009) DNA looping provides stability and robustness to the bacteriophage lambda switch. Proc Natl Acad Sci USA 106 (20): 8101–8106. 10.1073/pnas.0810399106 19416825 PMC2686219

[26] Li GW, Berg OG, Elf J (2009) Effects of macromolecular crowding and DNA looping on gene regulation kinetics. Nat Phys 5: 294–297.

[27] Anderson LM, Yang H (2008) DNA looping can enhance lysogenic CI transcription in phage lambda. Proc Natl Acad Sci USA 105 (15): 5827–5832. 10.1073/pnas.0705570105 18391225 PMC2311354

[28] Earnest TM, Roberts E, Assaf M, Dahmen K, Luthey-Schulten Z (2013) DNA looping increases the range of bistability in a stochastic model of the lac genetic switch. Phys Biol 10: 026002 10.1088/1478-3975/10/2/026002 23406725

[29] Boedicker JQ, Garcia HG, Phillips R (2013) Theoretical and experimental dissection of DNA loop-mediated repression. Phys Rev Lett 110: 018101 23383841 10.1103/PhysRevLett.110.018101 PMC3716456

[30] Vilar JMG, Saiz L (2014) Suppression and enhancement of transcriptional noise by DNA looping. Phys Rev E 89: 062703. 10.1103/PhysRevE.89.062703 25019810

[31] Vilar JMG, Leibler S (2003) DNA looping and physical constrains on transcriptional regulation. J Mol Biol 331: 981–989. 12927535 10.1016/s0022-2836(03)00764-2

[32] Choudhary K, Oehler S, Narang A (2014) Protein Distributions from a Stochastic Model of the Operon of E. coli with DNA Looping Analytical solution and comparison with experiments. PLoS ONE 9(7): e102580 10.1371/journal.pone.0102580 25055040 PMC4108355

[33] Doyle B, Fudenberg G, Imakaev M, Mirny LA (2014) Chromatin loops as allosteric modulators of enhancer-promoter interactions. PLoS Comput Biol 10(10): e1003867 10.1371/journal.pcbi.1003867 25340767 PMC4207457

[34] Larson DR (2011). What do expression dynamics tell us about the mechanism of transcription? Curr Opin Genet Dev 21:591–599. 10.1016/j.gde.2011.07.010 21862317 PMC3475196

[35] Kepler TB, Elston TC (2001). Stochasticity in transcriptional regulation: origins, consequences, and mathematical representations. Biophys J 81:3116–3036. 11720979 10.1016/S0006-3495(01)75949-8 PMC1301773

[36] Paulsson J (2005) Models of stochastic gene expression. Phys Life Rev 2:157–175.

[37] Sánchez A, Garcia HG, Jones D, Phillips R, Kondev J (2011). Effect of promoter architecture on the cell-to-cell variability in gene expression. PLoS Comput Biol 7:e1001100 10.1371/journal.pcbi.1001100 21390269 PMC3048382

[38] Sánchez A, Choubey S, Kondev J (2013) Stochastic models of transcription: From single molecules to single cells. Methods S1046-2023:00095–9. 10.1016/j.ymeth.2013.03.026 23557991

[39] Zhang JJ, Huang LF and Zhou TS (2014) Comment on ‘Binomial moment equations for chemical reaction networks’. Phys Rev Lett 112: 088901.

[40] Dekker J, Rippe K, Dekker M, Kleckner N (2002). Capturing chromosome conformation. Science 295: 1306–1311. 11847345 10.1126/science.1067799

[41] Lieberman-Aiden E, van Berkum NL, Williams L, Imakaev M, Ragoczy T, et al (2009). Comprehensive mapping of long-range interactions reveals folding principles of the human genome. Science 326: 289–293. 10.1126/science.1181369 19815776 PMC2858594

[42] Shen Y, Yue F, McCleary DF, Ye Z, Edsall L, et al (2012) A map of the cis-regulatory sequences in the mouse genome. Nature 488(7409):116–120. 10.1038/nature11243 22763441 PMC4041622

[43] Nord AS, Blow MJ, Attanasio C, Akiyama JA, Holt A, et al (2013) Rapid and pervasive changes in genome-wide enhancer usage during mammalian development. Cell 155(7):1521–1531. 10.1016/j.cell.2013.11.033 24360275 PMC3989111

[44] Tolhuis B, Palstra RJ, Splinter E, Grosveld F, de Laat W (2002) Looping and interaction between hypersensitive sites in the active beta-globin locus. Mol Cell 10(6):1453–1465. 12504019 10.1016/s1097-2765(02)00781-5

[45] Palstra RJ, Tolhuis B, Splinter E, Nijmeijer R, Grosveld F, de Laat W (2003) The β-globin nuclear compartment in development and erythroid differentiation. Nat Genet 35:190–194. 14517543 10.1038/ng1244

[46] Savitskaya E, Melnikova L, Kostuchenko M, Kravchenko E, Pomerantseva E, et al (2006). Study of long-distance functional interactions between Su(Hw) insulators that can regulate enhancer-promoter communication in Drosophila melanogaster. Mol Cell Biol, 26(3): 754–761. 16428433 10.1128/MCB.26.3.754-761.2006 PMC1347022

[47] Wang JC, Giaever GN (1988) Action at a distance along a DNA. Science 240(4850): 300–304. 3281259 10.1126/science.3281259

[48] Blackwood EM, Kadonaga JT (1998) Going the distance: a current view of enhancer action. Science 281(5373): 60–63. 9679020 10.1126/science.281.5373.60

[49] Dean A (2006) On a chromosome far, far away: LCRs and gene expression. Trends in Genet 22(1):38–45. 16309780 10.1016/j.tig.2005.11.001

[50] Liu Z, Scannell DR, Eisen MB, Tjian R (2011) Control of embryonic stem cell lineage commitment by core promoter factor, TAF3. Cell 146: 720–731. 10.1016/j.cell.2011.08.005 21884934 PMC3191068

[51] Kagey MH, Newman JJ, Bilodeau S, Zhan Y, Orlando DA (2010) Mediator and cohesion connect gene expression and chromatin architecture. Nature 467: 430–435. 10.1038/nature09380 20720539 PMC2953795

[52] Hsieh CL, Fei T, Chen YW, Li TT, Gao YF (2014) Enhancer RNAs participate in androgen receptor-driven looping that selectively enhances gene activation. Proc Natl Acad Sci USA 111: 7319–7324. 10.1073/pnas.1324151111 24778216 PMC4034202

[53] Tuan D, Kong S, Hu K (1992) Transcription of the hypersensitive site HS2 enhancer in erythroid cells. Proc Natl Acad Sci USA 89: 11219–11223. 1454801 10.1073/pnas.89.23.11219 PMC50521

[54] Chen J, Zhang ZJ, Li L, Chen BC, Revyakin A (2014) Single-molecule dynamics of enhancerosome assembly in embryonic stem cells. Cell 156: 1274–1285. 10.1016/j.cell.2014.01.062 24630727 PMC4040518

[55] Bulger M, Groudine M (1999) Looping versus linking: toward a model for long-distance gene activation. Genes Dev 13: 2465–2477. 10521391 10.1101/gad.13.19.2465

[56] Dorsett D (1999) Distant liaisons: long-range enhancer-promoter interactions in Drosophila. Curr Opin Genet Dev 9: 505–514. 10508687 10.1016/s0959-437x(99)00002-7

[57] Hatzis P, Talianidis I (2002) Dynamics of enhancer-promoter communication during differentiation-induced gene activation. Mol Cell 10: 1467–1477. 12504020 10.1016/s1097-2765(02)00786-4

[58] Blackwood EM, Kadonaga JT (1998) Going the distance: a current view of enhancer action. Science 281: 60–63. 9679020 10.1126/science.281.5373.60

[59] Wang QB, Carroll JS, Brown M (2005) Spatial and temporal recruitment of androgen receptor and its coactivators involves chromosomal looping and polymerase tracking. Mol Cell 19: 631–642. 16137620 10.1016/j.molcel.2005.07.018

[60] Lai F, Gardini A, Zhang AD, Shiekhattar R (2015) Integrator mediates the biogenesis of enhancer RNAs. Nature 525: 399–403. 10.1038/nature14906 26308897 PMC4718573

[61] Bintu L, Buchler NE, Garcia HG, Gerland U, Hwa T, et al (2005) Transcriptional regulation by the numbers: applications. Curr Opin Genet Dev 15: 125–135. 15797195 10.1016/j.gde.2005.02.006 PMC3462814

[62] Zhang JJ, Zhou TS (2014) Promoter-mediated transcriptional dynamics. Biophys. J. 106: 479–488. 10.1016/j.bpj.2013.12.011 24461023 PMC3907263

[63] Slater LJ (1960) Confluent Hypergeometric Functions. Cambridge University Press, Cambridge.

[64] Jonkers I, Lis JT (2015) Getting up to speed with transcription elongation by RNA polymerase II. Nat Rev Mol Cell Biol 16: 167–177. 10.1038/nrm3953 25693130 PMC4782187

[65] Spicuglia S, Kumar S, Yeh JH, Vachez E, Chasson L, et al (2002) Promoter activation by enhancer-dependent and -independent loading of activator and coactivator complexes. Mol Cell 10: 1479–1487. 12504021 10.1016/s1097-2765(02)00791-8

[66] Ho YG, Elefant F, Liebhaber SA, Cooke NE (2006) Locus control region transcription plays an active role in long-range gene activation. Mol Cell 23: 365–375. 16885026 10.1016/j.molcel.2006.05.041

[67] Zhao H, Friedman RD, Fournier REK (2007) The locus control region activates serpin gene expression through recruitment of liver-specific tran-scription factors and RNA polymerase II. Mol Cell Biol 27: 5286–5295. 17526725 10.1128/MCB.00176-07 PMC1952087

[68] Ghavi-Helm Y, Klein FA, Pakozdi T, Ciglar L, Noordermeer D, et al (2014) Enhancer loops appear stable during development and are associated with paused polymerase. Nature 512: 96–100. 10.1038/nature13417 25043061

[69] Szutorisz H, Georgiou A, Tora L, Dillon N, (2006) The proteasome restricts permissive transcription at tissue-specific gene loci in embryonic stem cells. Cell 127: 1375–1388. 17190601 10.1016/j.cell.2006.10.045

[70] Vernimmen D, Marques-Kranc F, Sharpe JA, Sloane-Stanley JA, Woo WG, et al (2009) Chromosome looping at the human alpha-globin locus is mediated via the major upstream regulatory element (HS-40). Blood 114: 4253–4260. 10.1182/blood-2009-03-213439 19696202

[71] Sawado T, Halow J, Bender MA, Groudine M (2003) The beta-globin locus control region (LCR) functions primarily by enhancing the transition from transcription initiation to elongation. Genes Dev 17: 1009–1018. 12672691 10.1101/gad.1072303 PMC196035

[72] Song SH, Kim A, Ragoczy Bender TMA, Groudine M, Dea A (2010) Multiple functions of Ldb1 required for beta-globin activation during erythroid differentiation. Blood 116: 2356–2364. 10.1182/blood-2010-03-272252 20570862 PMC2953839

[73] Bender MA, Ragoczy T, Lee J, Byron R, Telling A et al (2012) The hypersensitive sites of the murine beta-globin locus control region act independently to affect nuclear localization and transcriptional elongation. Blood 119: 3820–3827. 10.1182/blood-2011-09-380485 22378846 PMC3335386

[74] Lin CQ, Garruss AS, Luo ZJ, Guo FL, Shilatifard A (2013) The RNA Pol II elongation factor Ell3 marks enhancers in ES cells and primes future gene activation. Cell 152: 144–156. 10.1016/j.cell.2012.12.015 23273992 PMC3556173

[75] Schaukowitch K, Joo J-Y, Liu XH, Watts JK, Martinez C, Kim T-K (2014) Enhancer RNA facilitates NELF release from immediate early genes. Mol Cell 56: 29–42. 10.1016/j.molcel.2014.08.023 25263592 PMC4186258

[76] Seenundun S, Rampalli S, Liu QC, Aziz A, Palii C, et al (2010) UTX mediates demethylation of H3K27me3 at muscle-specific genes during myogenesis. EMBO J 29: 1401–1411. 10.1038/emboj.2010.37 20300060 PMC2868576

[77] Vernimmen D, Lynch MD, Gobbi MD, Garrick D, Sharpe JA, et al (2011) Polycomb eviction as a new distant enhancer function. Genes Dev 25: 1583–1588. 10.1101/gad.16985411 21828268 PMC3182025

[78] Taberlay PC, Kelly TK, Liu CC, You JS, de Carvalho DD, Miranda TB, et al (2011) Polycomb-repressed genes have permissive enhancers that initiate reprogramming. Cell 147: 1283–1294. 10.1016/j.cell.2011.10.040 22153073 PMC3240866

[79] Kondo T, Isono T, Kondo K, Endo TA, Itohara S, et al (2014) Polycomb potentiates meis2 activation in midbrain by mediating interaction of the promoter with a tissue-specific enhancer. Dev Cell 28: 94–101. 10.1016/j.devcel.2013.11.021 24374176

[80] Vernimmen D (2014) Uncovering enhancer functions using the alpha-globin locus. PLoS Genet 10: e1004668 10.1371/journal.pgen.1004668 25330308 PMC4199490

[81] Rippe K (2001) Making contacts on a nucleic acid polymer. Trends in Biochem Sci 26: 733–740. 11738597 10.1016/s0968-0004(01)01978-8

[82] Gondor A, Ohlsson R (2009) Chromosome crosstalk in three dimensions. Nature 461: 212–217. 10.1038/nature08453 19741702

[83] Juven-Gershon T, Kadonaga JT (2010) Regulation of gene expression via the core promoter and the basal transcrip-tional machinery. Dev Biol 339: 225–229. 10.1016/j.ydbio.2009.08.009 19682982 PMC2830304

[84] Muller F, Tora L (2014) Chromatin and DNA sequences in defining promoters for transcription initiation. Biochim. Biophys Acta 1839: 118–128 3. 10.1016/j.bbagrm.2013.11.003 24275614

[85] Butler JE, Kadonaga JT (2001) Enhancer–promoter specificity mediated by DPE or TATA core promoter motifs. Genes Dev 15: 2515–2519 6. 11581157 10.1101/gad.924301 PMC312797

[86] Zabidi MA, Arnold CD, Schernhuber K, Pagani M, Rath M, et al (2015) Enhancer-core-promoter specificity separates developmental and housekeeping gene regulation. Nature 518: 556–559. 10.1038/nature13994 25517091 PMC6795551

[87] Carter D, Chakalova L, Osborne CS, Dai YF, Frase P (2002) Long-range chromatin regulatory interactions in vivo. Nat Genet 32: 623–626. 12426570 10.1038/ng1051

[88] Tolhuis B, Palstra R-J, Splinter E, Grosveld F, de Laat W (2002) Looping and interaction between hypersensitive sites in the active b-globin locus. Mol Cell 10: 1453–1465. 12504019 10.1016/s1097-2765(02)00781-5

