# Supplementary material for: Effect of Interaction between Chromatin Loops on Cell-to-Cell Variability in Gene Expression
Source: PLoS Comput Biol. 2016 May 6;12(5):e1004917. doi: 10.1371/journal.pcbi.1004917 (PMC4859557; doi:10.1371/journal.pcbi.1004917)
Supplement: S1 Text — The former gives details for derivation of mathematical formulae used in the main text. The latter contains the following contents: (1) Three–dimensional effects for dependence of the mRNA mean/the mRNA noise on loop distance and tracking ratio; (2) Time evolution and distribution of the mRNA number; (3) Skewness and kurtosis of the mRNA distribution; (4) Effect of different transcript rates on gene expression; Effect of three interacting DNA loops on gene expression. (DOCX) [file pcbi.1004917.s001.docx]

**SUPPORTING INFORMATION**

**Effect of Interaction between Chromatin Loops on Cell-to-Cell Variability in Gene Expression**

Tuoqi Liu, Jiajun Zhang and Tianshou Zhou

School of Mathematics and Computational Science, Sun Yat-Sen University, Guangzhou 510275 P.R. China

**S1. Derivation of analytical results**

We begin by considering a general gene model described by Eq. (6) in the main text. Introduce factorial binomial moments for factorial distributions $P_{i}\left(m;t\right)$ with $i=1,2,\cdots,N$ and $N$ representing the total number of promoter activity states

$b_{k}^{\left(i\right)}=\sum_{m=k}^{\infty} \left(\begin{matrix} m \\ k \end{matrix}\right)\text{ }P_{i}\left(m;t\right)$, $k=0,1,2,\cdots$ (1)

Then, it is not difficult to derive the following equations from Eq. (6) in the main text

$\frac{d}{dt}b_{k}\left(t\right)=Ab_{k}\left(t\right)+\Lambda b_{k-1}\left(t\right)-k\delta\text{ }b_{k}\left(t\right)$ (2)

where $b_{k}=\left(b_{k}^{\left(1\right)},\cdots,b_{k}^{\left(N\right)}\right)^{T}$, $k=0,1,2,\cdots$. Denote by $b_{k}=\sum_{i=1}^{N} b_{k}^{\left(i\right)}$ the total binomial moment. Note that $\sum_{i=1}^{N} b_{0}^{\left(i\right)}\left(t\right)\equiv1$ for any time $t$ due to the conservative condition of the total probability defined as $P\left(m;t\right)=\sum_{i=1}^{N} P_{i}\left(m;t\right)$, i.e., due to $\sum_{m=0}^{\infty} P\left(m;t\right)\equiv1$ for any $t$. If we introduce the generating function $G\left(z;t\right)=\sum_{m} z^{m}P\left(m;t\right)$ for $P\left(m;t\right)$, then it is also not difficult to derive

$b_{k}\left(t\right)\equiv\frac{1}{k!}\left.\frac{\partial^{k}G\left(z;t\right)}{\partial z^{k}}\right|_{z=1}$, $k=0,1,2,\cdots$ (3)

according to the definition of $b_{k}=\sum_{m=k}^{\infty} \left(\begin{matrix} m \\ k \end{matrix}\right)\text{ }P\left(m;t\right)$. This is the relationship between binomial moments and the underlying generating function. It is well known that

$P\left(m;t\right)=\frac{1}{m!}\left.\frac{\partial^{m}G\left(z;t\right)}{\partial z^{m}}\right|_{z=0}$, $m=1,2,\cdots$ (4)

which shows how the generating function is used to give the corresponding distribution. Combing Eq. (4) with expression $G\left(z;t\right)=\sum_{k} b_{k}\left(t\right)\left(z-1\right)^{k}$ that is a power series of $G\left(z;t\right)$ expanded at $z=1$, we can derive a formula for reconstructing $P\left(m;t\right)$ from the binomial moments $b_{k}\left(t\right)$, i.e., Eq. (11) in the main text.

In general, steady-state distribution is the main interest. At steady state, it follows from Eq. (2) that

$b_{k}=\frac{1}{\prod_{i=1}^{k} \det\left(i\delta-\mathbf{A}\right)}\prod_{i=k}^{1} \left[\mathbf{u}_{N}\left(i\delta-\mathbf{A}\right)^{*}\Lambda\right]\text{ }b_{0}\begin{matrix} , & k=1,2,\cdots\end{matrix}$ (5)

where $\mathbf{u}_{N}=\left(\underbrace{1,1,\cdots,1}_{N}\right)$ is an $N-$dimensional row vector, the symbols $\left(i\delta-\mathbf{A}\right)^{*}$ and $\det\left(i\delta-\mathbf{A}\right)$ are respectively the adjacency matrix and the determinant of matrix $\left(i\delta-\mathbf{A}\right)$, and $b_{0}$ is a constant vector depending on system parameters. Note that if all the binomial moments $b_{k}$ are found either analytically or numerically by Eq. (5), the steady-state mRNA distribution can be calculated according to Eq. (11) in the main text. Therefore, the left question is how the vector $b_{0}$ is expressed using the system parameters. The following analysis is limited to the case that all the degradation rates are the same and are assumed as 1 without loss of generality (otherwise, it is needed to normalize system parameters by the common degradation rate).

Note that $A$is an M-matrix (i.e., the sum of every column elements is equal to zero). Therefore, we can assume the eigenvalues of matrix $A$ as $0,-\alpha_{1},-\alpha_{2},\cdots,-\alpha_{N-1}$ with $\alpha_{i}\neq0$ and the eigenvalues of sub-matrix $\mathbf{M}_{k}$ (i.e., the minor of the element $a_{kk}$ of matrix $A$) as $-\beta_{1}^{\left(k\right)},-\beta_{2}^{\left(k\right)},\cdots,-\beta_{N-1}^{\left(k\right)}$ with $\beta_{i}^{\left(k\right)}\neq0$. With this assumption, the characteristic polynomial of $A$ and $\mathbf{M}_{k}$ takes the form respectively

$f_{\mathbf{A}}\left(n\right)=\det\left(n\mathbf{I}_{N}-\mathbf{A}\right)=n\left(n+\alpha_{1}\right)\cdots\left(n+\alpha_{N-1}\right)$ (6)

$f_{\mathbf{M}_{k}}\left(n\right)=\det\left(n\mathbf{I}_{N-1}-\mathbf{M}_{k}\right)=\left(n+\beta_{1}^{\left(k\right)}\right)\cdots\left(n+\beta_{N-1}^{\left(k\right)}\right)$ (7)

As a consequence of [Laplace's formula](http://en.wikipedia.org/wiki/Laplace_expansion) for the determinant of matrix $A$, we have

$\mathbf{AA}^{*}=\det\left(\mathbf{A}\right)\mathbf{I}_{N}=\mathbf{0}\Rightarrow\mathbf{A}\left(\begin{matrix} \det\left(\mathbf{M}_{1}\right) \\ \vdots\\ \det\left(\mathbf{M}_{N}\right) \end{matrix}\right)=\mathbf{0}$, and $\mathbf{A}b_{0}=\mathbf{0}$ (8)

Since $rank\left(\mathbf{A}\right)=N-1$, the null space of $A$ is of one dimensional. Thus, we can set $b_{0}=c\left(\begin{matrix} \det\left(\mathbf{M}_{1}\right) \\ \vdots\\ \det\left(\mathbf{M}_{N}\right) \end{matrix}\right)$. From $\mathbf{u}_{N}b_{0}=1$ with $\mathbf{u}_{N}=\left(1,1,\cdots,1\right)$, we obtain

$c=\left[\sum_{k=1}^{N} \det\left(\mathbf{M}_{k}\right)\right]^{-1}=\left(-1\right)^{N-1}\left[\sum_{k=1}^{N} \prod_{i=1}^{N-1} \beta_{i}^{\left(k\right)}\right]^{-1}$ (9)

Note that from the expression of $f_{\mathbf{A}}\left(x\right)$, we have $\left.\frac{df_{\mathbf{A}}\left(x\right)}{dx}\right|_{x=0}=\prod_{i=1}^{N-1} \alpha_{i}$. On the other hand, Jacobi's formula gives

$\frac{df_{\mathbf{A}}\left(x\right)}{dx}=\frac{d\det\left(x\mathbf{I}_{N}-\mathbf{A}\right)}{dx}=tr\left(\left(x\mathbf{I}_{N}-\mathbf{A}\right)^{*}\frac{d\left(x\mathbf{I}_{N}-\mathbf{A}\right)}{dx}\right)=tr\left(\left(x\mathbf{I}_{N}-\mathbf{A}\right)^{*}\right)$ (10)

where “tr” represents the trace of a matrix. It follows from Eq. (10) that we have

$\left.\frac{df_{\mathbf{A}}\left(x\right)}{dx}\right|_{x=0}=tr\left(\left(-\mathbf{A}\right)^{*}\right)=\left(-1\right)^{N-1}\sum_{k=1}^{N} \det\left(\mathbf{M}_{k}\right)=\sum_{k=1}^{N} \prod_{i=1}^{N-1} \beta_{i}^{\left(k\right)}=\prod_{i=1}^{N-1} \alpha_{i}$ (11)

Finally, we obtain the following explicit expression of $b_{0}^{\left(k\right)}$

$b_{0}^{\left(k\right)}=c\det\left(\mathbf{M}_{k}\right)=\left(-1\right)^{N-1}\left(\sum_{k=1}^{N} \prod_{i=1}^{N-1} \beta_{i}^{\left(k\right)}\right)^{-1}\left(-1\right)^{N-1}\prod_{i=1}^{N-1} \beta_{i}^{\left(k\right)}=\prod_{i=1}^{N-1} \frac{\beta_{i}^{\left(k\right)}}{\alpha_{i}},\text{ }1\leq k\leq N$ (12)

It is worth pointing out that we can also use binomial moments to express the noise intensity defined as the ration of variance over the square of mean of a distribution, denoted by $\eta$, the skewness of this distribution, denoted by $\gamma_{1}$, characterizing the degree of the asymmetry of the distribution around its mean, and the kurtosis, denoted by $\gamma_{2}$, measuring the degree of peakedness of the distribution. That is,

$\eta=\frac{\mu_{2}}{\mu_{1}^{2}}$, $\gamma_{1}=\frac{\mu_{3}}{\mu_{2}^{3/2}}$, $\gamma_{2}=\frac{\mu_{4}}{\mu_{2}^{2}}-3$ (13)

where

$\mu_{k}=\left(-b_{1}\right)^{k}+\sum_{i=0}^{k-1} \sum_{j=1}^{k-i} R\left(k,i,j\right)\left(j!\right)\left(b_{1}\right)^{i}b_{j}$ (14)

in which $R\left(k,i,j\right)=\left(-1\right)^{i}\left(\begin{matrix} k \\ i \end{matrix}\right)S\left(k-i,j\right)$ and $S\left(n,k\right)$ is the Stirling number of the second kind [1].

Then, we list biochemical reactions corresponding to our four-state model of gene expression. They are

, , ,

, ,

where transition rates $\lambda_{ij}$ between promoter activity states are functions of loop lengths along DNA lines (Figure 2 in the main text indicates how they depend on the lengths of which DNA loops), and we do not consider translational processes. Let $P_{1}\left(m;t\right)$, $P_{2}\left(m;t\right)$, $P_{3}\left(m;t\right)$ and $P_{4}\left(m;t\right)$ represent the probabilities that the mRNA has $m$ copies at $OFF_{1}$, $OFF_{2}$, $ON_{1}$ and $ON_{2}$ states and at time $t$. According to the above reactions, it is easy for us to write the master equation given by Eq. (5) in the main text.

Next, we apply the above general results to the model considered in the main text. Note that in this case, we have

$b_{k}=\left(\begin{matrix} b_{k}^{\left(1\right)} \\ b_{k}^{\left(2\right)} \\ b_{k}^{\left(3\right)} \\ b_{k}^{\left(4\right)} \end{matrix}\right)$, $\Lambda=\left(\begin{matrix} 0 & 0 & 0 & 0 \\ 0 & 0 & 0 & 0 \\ 0 & 0 & \mu_{1} & 0 \\ 0 & 0 & 0 & \mu_{2} \end{matrix}\right)$, $\delta=\left(\begin{matrix} \delta_{1} & 0 & 0 & 0 \\ 0 & \delta_{2} & 0 & 0 \\ 0 & 0 & \delta_{3} & 0 \\ 0 & 0 & 0 & \delta_{4} \end{matrix}\right)$ (15a)

$A=\left(\begin{matrix} -\left(\lambda_{14}+\lambda_{12}\right) & \lambda_{21} & \lambda_{41} & 0 \\ \lambda_{12} & -\left(\lambda_{21}+\lambda_{23}\right) & 0 & \lambda_{32} \\ \lambda_{14} & 0 & -\left(\lambda_{41}+\lambda_{43}\right) & \lambda_{43} \\ 0 & \lambda_{23} & \lambda_{43} & -\left(\lambda_{32}+\lambda_{34}\right) \end{matrix}\right)$ (15b)

where $\lambda_{ij}$ may be functions of loop lengths along DNA lines. Assume that two transcription rates are equal, i.e., $\mu_{1}=\mu_{2}=\mu$, and four degradation rates are also equal, i.e., $\delta_{1}=\delta_{2}=\delta_{3}=\delta_{4}=\delta$. Furthermore, we set $\delta\text{=1}$ without loss of generality. For convenience, we denote by

$$\left(iI-A\right)^{*}=\left(\begin{matrix} i+\lambda_{12}+\lambda_{14} & -\lambda_{21} & -\lambda_{41} & 0 \\ -\lambda_{12} & i+\lambda_{21}+\lambda_{23} & 0 & -\lambda_{32} \\ -\lambda_{14} & 0 & i+\lambda_{41}+\lambda_{43} & -\lambda_{34} \\ 0 & -\lambda_{23} & -\lambda_{43} & i+\lambda_{32}+\lambda_{34} \end{matrix}\right)^{\text{*}}=\left(\begin{matrix} W_{11}^{\left(i\right)} & W_{21}^{\left(i\right)} & W_{31}^{\left(i\right)} & W_{41}^{\left(i\right)} \\ W_{12}^{\left(i\right)} & W_{22}^{\left(i\right)} & W_{32}^{\left(i\right)} & W_{42}^{\left(i\right)} \\ W_{13}^{\left(i\right)} & W_{23}^{\left(i\right)} & W_{33}^{\left(i\right)} & W_{43}^{\left(i\right)} \\ W_{14}^{\left(i\right)} & W_{24}^{\left(i\right)} & W_{34}^{\left(i\right)} & W_{44}^{\left(i\right)} \end{matrix}\right)$$

the adjacency matrix of matrix $\left(iI-\mathbf{A}\right)$. Then, we have

$W_{31}^{\left(i\right)}=\left|\begin{matrix} -\lambda_{21} & -\lambda_{41} & 0 \\ i+\lambda_{21}+\lambda_{23} & 0 & -\lambda_{32} \\ -\lambda_{23} & -\lambda_{34} & i+\lambda_{32}+\lambda_{34} \end{matrix}\right|,W_{41}^{\left(i\right)}=-\left|\begin{matrix} -\lambda_{21} & -\lambda_{41} & 0 \\ i+\lambda_{21}+\lambda_{23} & 0 & -\lambda_{32} \\ 0 & i+\lambda_{41}+\lambda_{43} & -\lambda_{34} \end{matrix}\right|$ (16a)

$W_{32}^{\left(i\right)}=-\left|\begin{matrix} i+\lambda_{12}+\lambda_{14} & -\lambda_{41} & 0 \\ -\lambda_{12} & 0 & -\lambda_{32} \\ 0 & -\lambda_{43} & i+\lambda_{32}+\lambda_{34} \end{matrix}\right|,W_{42}^{\left(i\right)}=\left|\begin{matrix} i+\lambda_{12}+\lambda_{14} & -\lambda_{41} & 0 \\ -\lambda_{12} & 0 & -\lambda_{32} \\ -\lambda_{14} & i+\lambda_{41}+\lambda_{43} & -\lambda_{34} \end{matrix}\right|$ (16b)

$W_{33}^{\left(i\right)}=\left|\begin{matrix} i+\lambda_{12}+\lambda_{14} & -\lambda_{21} & 0 \\ -\lambda_{12} & i+\lambda_{21}+\lambda_{23} & -\lambda_{32} \\ 0 & -\lambda_{23} & i+\lambda_{32}+\lambda_{34} \end{matrix}\right|,W_{34}^{\left(i\right)}=-\left|\begin{matrix} i+\lambda_{12}+\lambda_{14} & -\lambda_{21} & -\lambda_{41} \\ -\lambda_{12} & i+\lambda_{21}+\lambda_{23} & 0 \\ 0 & -\lambda_{23} & -\lambda_{43} \end{matrix}\right|$ (16c)

$W_{43}^{\left(i\right)}=-\left|\begin{matrix} i+\lambda_{12}+\lambda_{14} & -\lambda_{21} & 0 \\ -\lambda_{12} & i+\lambda_{21}+\lambda_{23} & -\lambda_{32} \\ -\lambda_{14} & 0 & -\lambda_{34} \end{matrix}\right|\text{，}W_{44}^{\left(i\right)}=\left|\begin{matrix} i+\lambda_{12}+\lambda_{14} & -\lambda_{21} & -\lambda_{41} \\ -\lambda_{12} & i+\lambda_{21}+\lambda_{23} & 0 \\ -\lambda_{14} & 0 & i+\lambda_{41}+\lambda_{43} \end{matrix}\right|$ (16d)

In order to find the iterative law in Eq. (5), we perform the following calculations

$$\left(iI-A\right)^{*}\Lambda=\mu\left(\begin{matrix} W_{11}^{\left(i\right)} & W_{21}^{\left(i\right)} & W_{31}^{\left(i\right)} & W_{41}^{\left(i\right)} \\ W_{12}^{\left(i\right)} & W_{22}^{\left(i\right)} & W_{32}^{\left(i\right)} & W_{42}^{\left(i\right)} \\ W_{13}^{\left(i\right)} & W_{23}^{\left(i\right)} & W_{33}^{\left(i\right)} & W_{43}^{\left(i\right)} \\ W_{14}^{\left(i\right)} & W_{24}^{\left(i\right)} & W_{34}^{\left(i\right)} & W_{44}^{\left(i\right)} \end{matrix}\right)\left(\begin{matrix} 0 & 0 & 0 & 0 \\ 0 & 0 & 0 & 0 \\ 0 & 0 & 1 & 0 \\ 0 & 0 & 0 & 1 \end{matrix}\right)=\mu\left(\begin{matrix} 0 & 0 & W_{31}^{\left(i\right)} & W_{41}^{\left(i\right)} \\ 0 & 0 & W_{32}^{\left(i\right)} & W_{42}^{\left(i\right)} \\ 0 & 0 & W_{33}^{\left(i\right)} & W_{43}^{\left(i\right)} \\ 0 & 0 & W_{34}^{\left(i\right)} & W_{44}^{\left(i\right)} \end{matrix}\right)$$

$$\left(\begin{matrix} 0 & 0 & W_{31}^{\left(i\right)} & W_{41}^{\left(i\right)} \\ 0 & 0 & W_{32}^{\left(i\right)} & W_{42}^{\left(i\right)} \\ 0 & 0 & W_{33}^{\left(i\right)} & W_{43}^{\left(i\right)} \\ 0 & 0 & W_{34}^{\left(i\right)} & W_{44}^{\left(i\right)} \end{matrix}\right)\left(\begin{matrix} 0 & 0 & W_{31}^{\left(i-1\right)} & W_{41}^{\left(i-1\right)} \\ 0 & 0 & W_{32}^{\left(i-1\right)} & W_{42}^{\left(i-1\right)} \\ 0 & 0 & W_{33}^{\left(i-1\right)} & W_{43}^{\left(i-1\right)} \\ 0 & 0 & W_{34}^{\left(i-1\right)} & W_{44}^{\left(i-1\right)} \end{matrix}\right)=\left(\begin{matrix} 0 & 0 & W_{31}^{\left(i\right)}W_{33}^{\left(i-1\right)}\text{+}W_{41}^{\left(i\right)}W_{34}^{\left(i-1\right)} & W_{31}^{\left(i\right)}W_{43}^{\left(i-1\right)}\text{+}W_{41}^{\left(i\right)}W_{44}^{\left(i-1\right)} \\ 0 & 0 & W_{32}^{\left(i\right)}W_{33}^{\left(i-1\right)}\text{+}W_{42}^{\left(i\right)}W_{34}^{\left(i-1\right)} & W_{32}^{\left(i\right)}W_{43}^{\left(i-1\right)}\text{+}W_{42}^{\left(i\right)}W_{44}^{\left(i-1\right)} \\ 0 & 0 & W_{33}^{\left(i\right)}W_{33}^{\left(i-1\right)}\text{+}W_{43}^{\left(i\right)}W_{34}^{\left(i-1\right)} & W_{33}^{\left(i\right)}W_{43}^{\left(i-1\right)}\text{+}W_{43}^{\left(i\right)}W_{44}^{\left(i-1\right)} \\ 0 & 0 & W_{34}^{\left(i\right)}W_{33}^{\left(i-1\right)}\text{+}W_{44}^{\left(i\right)}W_{34}^{\left(i-1\right)} & W_{34}^{\left(i\right)}W_{43}^{\left(i-1\right)}\text{+}W_{44}^{\left(i\right)}W_{44}^{\left(i-1\right)} \end{matrix}\right)$$

and

$$\mathbf{u}_{N}\left[\left(iI-A\right)^{*}\Lambda\right]\left\{\left[\left(i-1\right)I-A\right]^{*}\Lambda\right\}=\mu^{2}\left(0,0,C\left(i\right)W_{33}^{\left(i-1\right)}\text{+}D\left(i\right)W_{43}^{\left(i-1\right)},C\left(i\right)W_{34}^{\left(i-1\right)}\text{+}D\left(i\right)W_{44}^{\left(i-1\right)}\right)$$

where

$C\left(i\right)=\sum_{j=1}^{4} W_{3j}^{\left(i\right)}=\left|\begin{matrix} i+\lambda_{12}+\lambda_{14}+\lambda_{41} & \lambda_{41}-\lambda_{21} & \lambda_{41} \\ -\lambda_{12} & i+\lambda_{21}+\lambda_{23} & -\lambda_{32} \\ \lambda_{43} & \lambda_{43}-\lambda_{23} & i+\lambda_{32}+\lambda_{43}+\lambda_{34} \end{matrix}\right|$ (17a)

$D\left(i\right)=\sum_{j=1}^{4} W_{4j}^{\left(i\right)}=\left|\begin{matrix} i+\lambda_{12}+\lambda_{14} & -\lambda_{21} & -\lambda_{41} \\ \lambda_{32}-\lambda_{12} & i+\lambda_{21}+\lambda_{23}+\lambda_{32} & \lambda_{32} \\ \lambda_{34}-\lambda_{14} & \lambda_{34} & i+\lambda_{34}+\lambda_{41}+\lambda_{34} \end{matrix}\right|$ (17b)

For $k=1$, Eq. (5) becomes

$b_{1}=\frac{\mu^{1}}{1!}\frac{C\left(1\right)b_{0}^{\left(3\right)}+D\left(1\right)b_{0}^{\left(4\right)}}{\left(1+\alpha_{1}\right)\left(1+\alpha_{2}\right)\left(1+\alpha_{3}\right)}$ (18) where

$b_{0}^{\left(3\right)}\text{=}\frac{\left|\begin{matrix} \lambda_{12}+\lambda_{14} & -\lambda_{21} & 0 \\ -\lambda_{12} & \lambda_{21}+\lambda_{23} & -\lambda_{32} \\ 0 & -\lambda_{23} & \lambda_{32}+\lambda_{34} \end{matrix}\right|}{\left|\begin{matrix} \lambda_{12}+\lambda_{21}+\lambda_{23} & \lambda_{12} & \lambda_{12}-\lambda_{32} \\ \lambda_{14} & \lambda_{14}+\lambda_{43}+\lambda_{41} & \lambda_{14}-\lambda_{34} \\ -\lambda_{23} & -\lambda_{43} & \lambda_{32}+\lambda_{34} \end{matrix}\right|}$ (18a) $b_{0}^{\left(4\right)}\text{=}\frac{\left|\begin{matrix} \lambda_{12}+\lambda_{14} & -\lambda_{21} & -\lambda_{41} \\ -\lambda_{12} & \lambda_{21}+\lambda_{23} & 0 \\ -\lambda_{14} & 0 & \lambda_{43}+\lambda_{41} \end{matrix}\right|}{\left|\begin{matrix} \lambda_{12}+\lambda_{21}+\lambda_{23} & \lambda_{12} & \lambda_{12}-\lambda_{32} \\ \lambda_{14} & \lambda_{14}+\lambda_{43}+\lambda_{41} & \lambda_{14}-\lambda_{34} \\ -\lambda_{23} & -\lambda_{43} & \lambda_{32}+\lambda_{34} \end{matrix}\right|}$ (18b)

$\left(i+\alpha_{1}\right)\left(i+\alpha_{2}\right)\left(i+\alpha_{3}\right)=\left|\begin{matrix} i+\lambda_{12}+\lambda_{21}+\lambda_{23} & \lambda_{12} & \lambda_{12}-\lambda_{32} \\ \lambda_{14} & i+\lambda_{14}+\lambda_{43}+\lambda_{41} & \lambda_{14}-\lambda_{34} \\ -\lambda_{23} & -\lambda_{43} & i+\lambda_{32}+\lambda_{34} \end{matrix}\right|$ (18c)

For $k=2$, Eq. (5) becomes

$b_{2}=\frac{\mu^{2}}{2!}\frac{\left[C\left(2\right)W_{33}^{\left(1\right)}\text{+}D\left(2\right)W_{34}^{\left(1\right)}\right]b_{0}^{\left(3\right)}+\left[C\left(2\right)W_{43}^{\left(1\right)}\text{+}D\left(2\right)W_{44}^{\left(1\right)}\right]b_{0}^{\left(4\right)}}{\left(1+\alpha_{1}\right)\left(i+\alpha_{2}\right)\left(1+\alpha_{3}\right)\left(2+\alpha_{1}\right)\left(2+\alpha_{2}\right)\left(2+\alpha_{3}\right)}$ (19)

which can be expressed as the linear combination of two terms of the form$\frac{\mu^{k}}{k!}\prod_{i=1}^{k} \frac{\left(i+\gamma_{1}\right)\left(i+\gamma_{2}\right)\left(i+\gamma_{3}\right)}{\left(i+\alpha_{1}\right)\left(i+\alpha_{2}\right)\left(i+\alpha_{3}\right)}$. In general, by mathematical induction, we can show

$b_{k}=\tilde{b}_{0}^{\left(3\right)}\frac{\mu^{k}}{k!}\prod_{i=1}^{k} \frac{\left(i+\gamma_{1}^{\left(1\right)}\right)\left(i+\gamma_{2}^{\left(1\right)}\right)\left(i+\gamma_{3}^{\left(1\right)}\right)}{\left(i+\alpha_{1}\right)\left(i+\alpha_{2}\right)\left(i+\alpha_{3}\right)}+\tilde{b}_{0}^{\left(4\right)}\frac{\mu^{k}}{k!}\prod_{i=1}^{k} \frac{\left(i+\gamma_{1}^{\left(2\right)}\right)\left(i+\gamma_{2}^{\left(2\right)}\right)\left(i+\gamma_{3}^{\left(2\right)}\right)}{\left(i+\alpha_{1}\right)\left(i+\alpha_{2}\right)\left(i+\alpha_{3}\right)}$ (20)

where all $\gamma$ are constants depending on system parameters, and $\tilde{b}_{0}^{\left(i\right)}\text{=}b_{0}^{\left(i\right)}/\left(b_{0}^{\left(3\right)}+b_{0}^{\left(4\right)}\right)$ with $i=3,4$. According to Eq. (11) in the main text, we thus obtain the following explicit expression of steady-state mRNA distribution

$P\left(m\right)=\tilde{b}_{0}^{\left(3\right)}P_{1}\left(m\right)+\tilde{b}_{0}^{\left(4\right)}P_{2}\left(m\right)$ (21)

where

$P_{i}\left(m\right)=\frac{\mu^{m}}{m!}\frac{\left(\gamma_{1}^{\left(i\right)}\right)_{m}\left(\gamma_{2}^{\left(i\right)}\right)_{m}\left(\gamma_{3}^{\left(i\right)}\right)_{m}}{\left(\alpha_{1}\right)_{m}\left(\alpha_{2}\right)_{m}\left(\alpha_{3}\right)_{m}}{}_{3}F_{3}\left(\left.\begin{matrix} m+\gamma_{1}^{\left(i\right)} & m+\gamma_{2}^{\left(i\right)} & m+\gamma_{3}^{\left(i\right)} \\ m+\alpha_{1} & m+\alpha_{2} & m+\alpha_{3} \end{matrix}\right|;-\mu\right),\text{ }i=1,2$ (21a)

in which ${}_{n}F_{n}\left(a_{1},\cdots,a_{n};b_{1},\cdots,b_{n};z\right)$ is a confluent hypergeometric function [2], and $\left(x\right)_{n}$ is the Pochhammer symbol and defined as $\left(x\right)_{n}=\Gamma\left(x+n\right)/\Gamma\left(x\right)$ with $\Gamma\left(x\right)$ being the common gamma function. Equation (21) indicates that the mRNA distribution is a linear combination of two distributions with each being a distribution in a two-state model of gene expression at the transcription level.

Next, we consider three particular cases. First, according to experimental evidence [3,4], we can set $\lambda_{14}=\lambda_{23}$, $\lambda_{12}=\lambda_{43}$, $\lambda_{41}=\lambda_{32}$, and $\lambda_{21}=\lambda_{34}$ for the side-by-side pattern, $0.5\lambda_{14}=\lambda_{23}$, $0.5\lambda_{12}=\lambda_{43}$, $\lambda_{41}=\lambda_{32}$, and $\lambda_{21}=\lambda_{\text{、}34}$ for the alternating pattern, and $\left(4e^{-0.5d}+1\right)\lambda_{14}=\lambda_{23}$, $\left(4e^{-0.5d}+1\right)\lambda_{12}=\lambda_{43}$, $\lambda_{41}=\lambda_{32}$, and $\lambda_{21}=\lambda_{34}$ for the nested pattern. For the side-by-side pattern, we further assume $\lambda_{21}=\lambda_{34}=\lambda_{32}=\lambda_{41}=\lambda$, and set $\lambda_{23}=k_{1}\lambda_{14},\text{ }\lambda_{43}=k_{2}\lambda_{12}$, then

$\begin{matrix} C\left(i\right)=\left|\begin{matrix} i+\lambda+\lambda_{12}+\lambda_{14} & 0 & \lambda\\ -\lambda_{12} & i+\lambda+k_{1}\lambda_{14} & -\lambda\\ k_{2}\lambda_{12} & k_{2}\lambda_{12}-k_{1}\lambda_{14} & i+2\lambda+k_{2}\lambda_{12} \end{matrix}\right|=\left|\begin{matrix} i+\lambda+\lambda_{12}+\lambda_{14} & 0 & \lambda\\ i+\lambda+\lambda_{14} & i+\lambda+k_{1}\lambda_{14} & 0 \\ \left(k_{2}-1\right)\lambda_{12} & i+\lambda+k_{2}\lambda_{12} & i+\lambda+k_{2}\lambda_{12} \end{matrix}\right| \\ =\lambda\left(i+\lambda+\lambda_{14}\right)\left(i+\lambda+k_{2}\lambda_{12}\right)+\left(i+\lambda+k_{1}\lambda_{14}\right)\left[\left(i+\lambda+\lambda_{12}+\lambda_{14}\right)\left(i+\lambda+k_{2}\lambda_{12}\right)-\left(k_{2}-1\right)\lambda\lambda_{12}\right] \end{matrix}$ (22a)

$\begin{matrix} D\left(i\right)=\left|\begin{matrix} i+\lambda_{12}+\lambda_{14} & -\lambda& -\lambda\\ \lambda-\lambda_{12} & i+2\lambda+k_{1}\lambda_{14} & \lambda\\ \lambda-\lambda_{14} & \lambda& i+2\lambda+k_{2}\lambda_{12} \end{matrix}\right|=\left|\begin{matrix} i+\lambda_{12}+\lambda_{14} & -\lambda& -\lambda\\ i+\lambda+\lambda_{14} & i+\lambda+k_{1}\lambda_{14} & 0 \\ i+\lambda+\lambda_{12} & 0 & i+\lambda+k_{2}\lambda_{12} \end{matrix}\right| \\ =\lambda\left(i+\lambda+\lambda_{14}\right)\left(i+\lambda+k_{2}\lambda_{12}\right)+\left(i+\lambda+k_{1}\lambda_{14}\right)\left[\left(i+\lambda_{12}+\lambda_{14}\right)\left(i+\lambda+k_{2}\lambda_{12}\right)+\lambda\left(i+\lambda+\lambda_{12}\right)\right] \end{matrix}$ (22b)

both being cubic polynomials of index $i$,

$b_{0}^{\left(3\right)}\text{=}\frac{\left|\begin{matrix} \lambda_{12}+\lambda_{14} & -\lambda& 0 \\ -\lambda_{12} & \lambda+k_{1}\lambda_{14} & -\lambda\\ 0 & -k_{1}\lambda_{14} & 2\lambda\end{matrix}\right|}{\left|\begin{matrix} \lambda+\lambda_{12}+k_{1}\lambda_{14} & \lambda_{12} & \lambda_{12}-\lambda\\ \lambda_{14} & \lambda+\lambda_{14}+k_{2}\lambda_{12} & \lambda_{14}-\lambda\\ -k_{1}\lambda_{14} & -k_{2}\lambda_{12} & 2\lambda\end{matrix}\right|}$ (22c)

$b_{0}^{\left(4\right)}\text{=}\frac{\left|\begin{matrix} \lambda_{12}+\lambda_{14} & -\lambda& -\lambda\\ -\lambda_{12} & \lambda+k_{1}\lambda_{14} & 0 \\ -\lambda_{14} & 0 & \lambda+k_{2}\lambda_{12} \end{matrix}\right|}{\left|\begin{matrix} \lambda+\lambda_{12}+k_{1}\lambda_{14} & \lambda_{12} & \lambda_{12}-\lambda\\ \lambda_{14} & \lambda+\lambda_{14}+k_{2}\lambda_{12} & \lambda_{14}-\lambda\\ -k_{1}\lambda_{14} & -k_{2}\lambda_{12} & 2\lambda\end{matrix}\right|}$ (22d)

both being constants depending on system parameters, and

$\left(i+\alpha_{1}\right)\left(i+\alpha_{2}\right)\left(i+\alpha_{3}\right)=\left|\begin{matrix} i+\lambda+\lambda_{12}+k_{1}\lambda_{14} & \lambda_{12} & \lambda_{12}-\lambda\\ \lambda_{14} & i+\lambda+\lambda_{14}+k_{2}\lambda_{12} & \lambda_{14}-\lambda\\ -k_{1}\lambda_{14} & -k_{2}\lambda_{12} & i+2\lambda\end{matrix}\right|$ (22e)

that is also a cubic polynomial of index $i$. We do not tend to expand these determinants due to complexity. In spite of this, the steady-state mRNA distribution is still given by Eq. (21). In Eqs. (22a)-(22e), $\lambda_{12}$ and $\lambda_{14}$ are functions of loop lengths, given by Eq. (1) in the main text.

Second, on the basis of the above assumption, we further assume $\lambda_{12}=\lambda_{14}=\lambda$ and $k_{1}=k_{2}=k$. In this case, we have

$C\left(i\right)=\left(i+\lambda+k\lambda\right)\left[i^{2}+\left(k+4\right)\lambda i+\left(k+5\right)\lambda^{2}\right]$ (23a)

$D\left(i\right)=\left(i+2\lambda\right)\left(i+\lambda+k\lambda\right)\left(i+3\lambda+k\lambda\right)$ (23b)

$\left(i+\alpha_{1}\right)\left(i+\alpha_{2}\right)\left(i+\alpha_{3}\right)=\left(i+2\lambda\right)\left(i+\lambda+k\lambda\right)\left(i+3\lambda+k\lambda\right)$ (23c)

$b_{0}^{\left(3\right)}\text{=}\frac{1}{k+3},\text{ }b_{0}^{\left(4\right)}=\frac{k}{k+3}$ (23d)

$W_{33}^{\left(i\right)}=\left(i+2\lambda\right)\left[i^{2}+\left(k+3\right)\lambda i+\left(k+1\right)\lambda^{2}\right],\text{ }W_{34}^{\left(i\right)}=k\lambda\left(i+2\lambda\right)\left(i+\lambda+k\lambda\right)$ (23e)

$W_{43}^{\left(i\right)}=\lambda\left(i+2\lambda\right)\left(i+\lambda+k\lambda\right)\text{, }W_{44}^{\left(i\right)}=\left(i+\lambda+k\lambda\right)\left[i^{2}+\left(k+3\right)\lambda i+2k\lambda^{2}\right]$ (23f)

Therefore, we can further show

$\mathbf{u}_{N}\left[\left(iI-A\right)^{*}\Lambda\right]\left\{\left[\left(i-1\right)I-A\right]^{*}\Lambda\right\}b_{0}=\frac{\tilde{\mu}^{2}}{i\left(i-1\right)}\frac{\left(a_{1}+i-1\right)\left(a_{1}+i-2\right)\left(a_{2}+i-1\right)\left(a_{2}+i-2\right)}{\left(b_{1}+i-1\right)\left(b_{1}+i-2\right)\left(b_{2}+i-1\right)\left(b_{2}+i-2\right)}$ (24)

where $a_{i}$, $b_{i}$ and $\tilde{\mu}$ are constants but depend on system parameters including parameter $k$. This implies that

$b_{k}=\frac{\tilde{\mu}^{k}}{k!}\frac{\left(a_{1}\right)_{k}\left(a_{2}\right)_{k}}{\left(b_{1}\right)_{k}\left(b_{2}\right)_{k}}$ (25)

Substituting Eq. (24) into Eq. (11) in the main text yields

$P\left(m\right)=\frac{\tilde{\mu}^{m}}{m!}\frac{\left(a_{1}\right)_{m}\left(a_{2}\right)_{m}}{\left(b_{1}\right)_{m}\left(b_{2}\right)_{m}}{}_{2}F_{2}\left(\left.\begin{matrix} m+a_{1} & m+a_{2} \\ m+b_{1} & m+b_{2} \end{matrix}\right|;-\tilde{\mu}\right)$ (26)

This distribution is similar to that in a two-state model of stochastic transcription [5]. With Eq. (25), it is easy to calculate the corresponding noise intensity according to Eq. (12) in the main text.

Third, on the basis of the above assumption, we further assume $k_{1}=k_{2}=1$, i.e., all the transition rates are the same. In this case, the steady-state binomial moment equations become

$\left(\begin{matrix} i+2\lambda& -\lambda& -\lambda& 0 \\ -\lambda& i+2\lambda& 0 & -\lambda\\ -\lambda& 0 & i+2\lambda& -\lambda\\ 0 & -\lambda& -\lambda& i+2\lambda\end{matrix}\right)\left(\begin{matrix} b_{k}^{\left(1\right)} \\ b_{k}^{\left(2\right)} \\ b_{k}^{\left(3\right)} \\ b_{k}^{\left(4\right)} \end{matrix}\right)=\mu\left(\begin{matrix} 0 & 0 & 0 & 0 \\ 0 & 0 & 0 & 0 \\ 0 & 0 & 1 & 0 \\ 0 & 0 & 0 & 1 \end{matrix}\right)\left(\begin{matrix} b_{k-1}^{\left(1\right)} \\ b_{k-1}^{\left(2\right)} \\ b_{k-1}^{\left(3\right)} \\ b_{k-1}^{\left(4\right)} \end{matrix}\right)$ (27)

Owing to symmetry of this equation (implying that $b_{k}^{\left(1\right)}=b_{k}^{\left(2\right)}$and $b_{k}^{\left(3\right)}=b_{k}^{\left(4\right)}$ for any $k$) and noting $b_{0}=b_{0}^{\left(1\right)}+b_{0}^{\left(2\right)}+b_{0}^{\left(3\right)}+b_{0}^{\left(4\right)}=1$, we obtain

$b_{k}^{\left(1\right)}=b_{k}^{\left(2\right)}=b_{0}^{\left(3\right)}\frac{\mu^{k}}{k\text{！}}\prod_{i=1}^{k} \frac{i-1+\lambda}{i+2\lambda}$ (28)

$b_{k}^{\left(3\right)}=b_{k}^{\left(4\right)}=b_{0}^{\left(3\right)}\frac{k+\lambda}{\lambda}\frac{\mu^{k}}{k\text{！}}\prod_{i=1}^{k} \frac{i-1+\lambda}{i+2\lambda}$ (29)

where $b_{0}^{\left(3\right)}=b_{0}^{\left(4\right)}=1/4$. Thus,

$b_{k}=2b_{k}^{\left(1\right)}+2b_{k}^{\left(3\right)}=\frac{k+2\lambda}{2\lambda}\frac{\mu^{k}}{k\text{！}}\prod_{i=1}^{k} \frac{i-1+\lambda}{i+2\lambda}=\frac{\mu^{k}}{k\text{！}}\prod_{i=0}^{k-1} \frac{i+\lambda}{i+2\lambda}=\frac{\mu^{k}}{k\text{！}}\frac{\left(\lambda\right)_{k}}{\left(2\lambda\right)_{k}}$ (30)

$P\left(m\right)=\frac{\mu^{m}}{m!}\frac{\left(\lambda\right)_{m}}{\left(2\lambda\right)_{m}}{}_{1}F_{1}\left(m+\lambda,m+2\lambda;-\mu\right)$ (31)

It is easy to calculate the corresponding noise intensity also according to Eq. (12) in the main text.

**S1. Supplementary numerical results**

This supplementary section contains: (1) 3-D pseudo diagrams for the dependence of the mean RNA level and the expression noise on loop distance and tracking ratio (see definition in the main text); (2) time evolutions and distributions of the mRNA number; and (3) skewness and kurtosis of the mRNA distribution. The aim is twofold: (1) enriching the content on the effect of interacting DNA loops on gene expression; (2) showing advantages of our binomial moment approach.

**S1.1 Three-dimensional effects for dependence of the mRNA mean/the mRNA noise on loop distance and tracking ratio**

**Figure A.** **Dependence of the mean mRNA level (A) and the mRNA noise intensity (B) on the loop distance** (horizontal axis) **and the tracking ratio** (ordinate axis). This full figure corresponds to Fig. 4 in the main text (meaning that the parameter values are the same).

**Figure B.** **Dependence of the mean mRNA level (A) and the mRNA noise intensity (B) on the green loop distance** (horizontal axis) **and the tracking ratio** (ordinate axis). This full figure corresponds to Figure **5** in the main text (meaning that the parameter values are the same).

**S1.2 Time evolution and distribution of the mRNA number**

The following Figures **C**-**H** show the time evolutions of and the distributions of the mRNA number. These figures correspond to Figure 3 in the main text.

**Figure C.** **Time evolutions** (left) **and distributions** (right) **of the mRNA number.** **alternating structure**. This figure corresponds to Figure **C (A)** and **C (B)** in the main text, but the blue loop distance is set as: (A) 10, (B) 60, and (C) 1000.

**Figure D.** **Time evolutions** (left) **and distributions** (right) **of the mRNA number.** **nested structure**. This figure corresponds to Figure **C (A)** and **C (B)** in the main text, but the blue loop distance is set as: (A) 10, (B) 20, and (C) 100.

**Figure E.** **Time evolutions** (left) **and distributions** (right) **of the mRNA number.** **alternating structure**. This full figure corresponds to Figure **C (A)** and **C (B)** in the main text, but the blue loop distance is set as: (A) 10 (A), (B) 60, and (C) 600.

**Figure F.** **Time evolutions** (left) **and distributions** (right) **of the mRNA number.** **nested structure**. This full figure corresponds to Figure **C (A)** and **C (B)** in the main text, but the blue loop distance is set as: (A) 10, (B) 60, and (C) 600.

The following Figures G and H show the time evolutions and the distributions of the mRNA number. These two figures correspond respectively to Figure **D** and Figure **E** in the main text.

**Figure G.** **Time evolutions** (left) **and distributions** (right) **of the mRNA number.** This full figure corresponds to Figure **D** in the main text: (A) no tracking (blue), and (B) tracking (red), but the green loop distance is set as 2000.

**Figure H.** **Time evolutions** (left) **and distributions** (right) **of the mRNA number.** This full figure corresponds to Figure **E** in the main text: (A) no tracking (blue), and (B) tracking (red), but the green loop distance is set as 2000.

**S1.3 Skewness and kurtosis of the mRNA distribution**

If a distribution is Poissonian, then the first two moments can well characterize this distribution. For many biochemical reaction networks, the corresponding distributions are non-Poissnian. In this case, it is needed to analyze kurtosis and skewness since the mean and the noise intensity are not enough to characterize the distributions. Here, we use the above formulae (13) to calculate the kurtosis and skewness of the mRNA distribution. The numerical results are shown in Figure **S9** and Figure **S10**. From these figures, we observe some different characteristics of kurtosis and skewness from those of mRNA mean and noise intensity. In addition, nonzero kurtosis and skewness imply that the distribution is non-Poissonian.

**Figure I.** **Skewness** (the first column) **and kurtosis** (the second column) **of the mRNA distribution**. This full figure corresponds to Figure **D** in the main text.

**Figure J.** **Skewness** (the first column) **and kurtosis** (the second column) **of the mRNA distribution**. This full figure corresponds to Figure **E** in the main text.

**S1.4 Effect of different transcription rates on gene expression**

**Figure K.** **Effect of different transcription rates on gene expression**, where $\mu_{1}\text{=10}$, and $\mu_{2}\text{=5}$ for alternating loops; $\mu_{1}\text{=10}$, and $\mu_{2}\text{=15}$ for nested loops; and $\mu_{1}\text{=10}$, and $\mu_{2}\text{=10}$ for side-by-side loops;. The other parameter values are set as those in Figure **C** in the main text.

**S1.5 Effect of three interacting DNA loops on gene expression**

Here we simply show how three interacting DNA loops (referring to Figure **L**) affect gene expression. The numerical results are demonstrated in Figure **M**. From this figure, we can conclude that more two interacting DNA loops do not change our main conclusion qualitatively. In other words, the qualitative results obtained in this paper do not depend on the number and the structure of interacting DNA loops.

**Figure L.** **Schematic diagram for three interacting DNA loops**.

**Figure M.** **Numerical results for the effect of three interacting DNA loops on gene expression**. Similar to the case of two interacting DNA loops, the main results are that (1) only for moderate loop distances, does the mean expression level or the noise intensity have apparent changes; (2) the dependence of the mean level on the loop distance is fundamentally opposite to that of the noise intensity on the loop distance.

**References**

1. Balakrishnan N, Johnson NL, Kotz S (1998) A note on relationships between moments, central moments and cumulants from multivariate distributions. Stat Probabil Lett 39: 49-54.
2. Slater LJ (1960) Confluent Hypergeometric Functions. Cambridge University Press, Cambridge.
3. Priesta DG, Kumar S, Yan Y, Dunlap DD, Dodda IB, Shearwin KE (2014). Quantitation of interactions between two DNA loops demonstrates loop domain insulation in E. coli cells. Proc Natl Acad Sci USA 111(42): E4449–E4457.
4. Doyle B, Fudenberg G, Imakaev M, Mirny LA (2014) Chromatin loops as allosteric modulators of enhancer-promoter interactions. PLoS Comput Biol 10(10): e1003867.
5. Zhou TS, Zhang JJ. Analytical results for a multi-state gene model. SIAM Journal on Applied Mathematics 72, 789-818 (2012).
